# Modelling diabetes and depression in Pakistan: using economic modelling to inform intervention design and a clinical trial of a behavioural activation intervention

**DOI:** 10.1136/bmjopen-2024-092158

**Published:** 2025-05-19

**Authors:** David Glynn, Pedro Saramago, Naveed Ahmed, Saima Afaq, Faiza Aslam, Abdul Basit, David Ekers, Asher Fawwad, Naomi Gibbs, Edward Fottrell, Richard Ian Gregory Holt, Rowena Jacobs, Asima Khan Niazi, Zia Ul-Haq, Gerardo A Zavala, Najma Siddiqi, Simon Walker

**Affiliations:** 1 Centre for Research in Medical Devices (CÚRAM) and Health Economics and Policy Analysis Centre, University of Galway, Ireland, Galway, Ireland; 2 University of York Centre for Health Economics, York, UK; 3 Centre for Health Research and Implementation, Diabetic Association of Bangladesh, Dhaka, Bangladesh; 4 BIRDEM General Hospital, Dhaka, Bangladesh; 5 Department of Health Sciences, University of York, York, UK; 6 Khyber Medical University, Peshawar, Pakistan; 7 Rawalpindi Medical University, Rawalpindi, Pakistan; 8 Department of Medicine, Baqai Institute of Diabetology and Endocrinology, Baqai Medical University, Karachi, Pakistan; 9 Tees Esk and Wear Valleys NHS Foundation Trust, Darlington, UK; 10 Institute for Global Health, University College London, London, UK; 11 Human Development and Health, Faculty of Medicine, University of Southampton, Southampton, UK

**Keywords:** Health economics, DIABETES & ENDOCRINOLOGY, MENTAL HEALTH

## Abstract

**Abstract:**

**Objectives:**

The ‘Developing and evaluating an adapted behavioural activation intervention for depression and diabetes in South Asia (DiaDeM)’ trial investigates a psychological intervention, behavioural activation (BA), on people with both diabetes and depression in Bangladesh and Pakistan. This study aimed to aid the intervention and trial design.

**Design:**

This was a modelling study using microsimulation to assess the intervention’s cost-effectiveness. Diabetes was modelled using the UK Prospective Diabetes Study model based on Pakistani patients and depression was modelled using Patient Health Questionnaire-9 (PHQ-9) trajectories allowing for multiple depressive episodes. It was assumed that diabetes-related adverse events increased depression recurrence, while depression impacted haemoglobin A1c, increasing diabetes-related events. The model estimated (1) maximum cost of BA which would be cost-effective (headroom analysis) to inform intervention design, and (2) value of reducing uncertainty around different measures (value of information analysis) to prioritise data collection in the DiaDeM study.

**Setting:**

Analysis was conducted from a Pakistani healthcare perspective over a lifetime with costs and outcomes discounted at 3%.

**Interventions:**

BA plus usual care was compared against usual care. BA involved six sessions by a trained (non-mental health) facilitator. The usual care comparator was the prevailing mix of pharmacological and non-pharmacological treatments used in Pakistan.

**Primary and secondary outcome measures:**

The primary outcome was disability-adjusted life-years (DALYs). Secondary outcomes included life years, healthcare costs and the rate of depression and diabetes-related events.

**Results:**

Over their lifetime, individuals receiving BA plus usual care avoid 3.2 (95% credible interval: 2.7 to 3.8) years of mild depression and experience fewer diabetes-related events. BA plus usual care resulted in an additional 0.27 (0.03 to 0.52) life years, 0.98 (0.45 to 1.86) DALYs averted and had incremental healthcare costs of −US$97 (−US$517 to US$142), excluding BA costs. The maximum cost per BA course at which was cost-effective is US$83 (US$9 to US$214). Value of information analysis found the most important measures to include in the trial are the impact of depression on diabetes and PHQ-9 over time.

**Conclusions:**

This is the first model to jointly model depression and diabetes for South Asia and uses novel methods to reflect the diseases and inform intervention and trial design. This evidence has helped to inform the design of the DiaDeM intervention and the trial to evaluate it.

**Trial registration:**

DiaDeM trial: ISRCTN40885204, DOI: ; pre-results, DOI: https://doi.org/10.1186/ISRCTN40885204, DiaDeM-NIHR200806

**STRENGTHS AND LIMITATIONS OF THIS STUDY**

- The study modelled the relationship between depression and diabetes to better capture the impact of the intervention of interest on both conditions.
- Depression was modelled as a recurrent, episodic condition, enhancing the reliability of results.
- The study captures the health outcomes and costs for individuals with diabetes and depression in Pakistan.
- Due to data limitations, the analysis was conducted for Pakistan only; however, the Developing and evaluating an adapted behavioural activation intervention for depression and diabetes in South Asia trial will be carried out in Pakistan and Bangladesh.
- Evidence was synthesised from diverse sources and settings, and this raises challenges around the comparability and generalisability to Pakistan.

## Introduction

 Individuals can experience multiple long-term conditions (MLTCs) simultaneously.^1 2^ The co-occurrence of diabetes and depression is recognised as an important issue.^3^,^5^ These conditions can interact in complex ways which can increase morbidity and mortality. MLTCs are increasingly recognised as an important issue globally which requires research.^6 7^

The ‘Developing and evaluating an adapted behavioural activation intervention for depression and diabetes in South Asia’ (DiaDeM) trial is designed to address the co-occurrence of these MLTCs.^8^ The DiaDeM trial is taking place in Bangladesh and Pakistan.^9^ It aims to compare adapted behavioural activation (BA) therapy delivered by non-specialist health workers in addition to optimised usual care to optimised usual care alone. BA is a psychological treatment that has been shown to treat depression effectively and can be delivered by non-specialist health workers. BA helps people make the link between what they do and how they feel and supports them to make changes to improve their health.^10 11^ However, evidence is largely from high-income countries and may not be generalisable to South Asian low-middle-income countries because of differences in the cultural context (including attitudes towards depression and talking therapies) and healthcare systems. Further, there is limited evidence on BA for the treatment of depression as part of MLTCs, including diabetes.^12^ This study will add to the growing literature on cost-effectiveness in the context of MLTCs.^7 13 14^

In this paper, we develop a novel decision analytical model and use it to inform the design of the DiaDeM intervention and trial.^15^ This model takes account of the natural history of diabetes and depression and any interactions. It predicts costs, morbidity and mortality over a patient’s lifetime, adjusting costs and patient characteristics to match the decision context. There were two aims of the analysis. First, conducting a headroom analysis to estimate the maximum cost of a BA treatment which would still be considered cost-effective for a given treatment effect.^16 17^ These helped to inform the number of BA sessions to provide. Second, conducting value of information (VOI) analyses to inform the trial design by estimating the relative value of collecting specific outcome measures during the trial, for example, should we prioritise collecting data on systolic blood pressure or low-density lipoprotein.^18 19^ Further, this model will be used to assess the long-term cost-effectiveness of the DiaDeM BA intervention following the culmination of the definitive trial.

## Methods

### Overview

An MLTC diabetes and depression decision analytical model was developed to estimate the lifetime cost and health impacts of a BA intervention in addition to usual care versus usual care alone in Pakistan for individuals with both diabetes and depression.^15^ This analysis focuses on Pakistan only because of a lack of data for Bangladesh. Health outcomes included life years and disability-adjusted life-years (DALYs) averted, a generic health outcome capturing morbidity and mortality. Costs reflected those related to healthcare, including out-of-pocket payments given the mixed public-private nature of the Pakistan healthcare system.^20^ Costs and outcomes were discounted at a rate of 3% per annum in line with international guidelines.^21^

The decision model was based on a previously developed diabetes model and an innovative de novo depression component.^1422^,^24^ All patients enter the model with known diabetes and depression. The treatment options modelled are those included in the DiaDeM trial: BA in addition to usual care versus ‘optimised’ usual care. The BA intervention is delivered in six face-to-face or online sessions by a trained (non-mental health) facilitator. The usual care comparator is the prevailing mix of pharmacological and non-pharmacological treatments used in Pakistan, ‘optimisation’ in this case is the provision of an information leaflet with details for accessing care locally.^25^

Next, we describe the diabetes model, then the depression model, and finally we describe the nature of their interaction. This model was developed in R, and the Viking computing cluster at the University of York was used to carry out all analyses.^26^

### Patient and public involvement

The model was produced in collaboration with local researchers and the international DiaDeM advisory group, which included academics, policy-makers and patient representatives.

### Diabetes model

The diabetes component of the model is based on the ‘UK Prospective Diabetes Study’ (UKPDS) Outcomes Model 2 which captures the risk of diabetes complications and mortality over an individual’s lifetime based on their characteristics including general characteristics (eg, age, gender, years with diabetes) and a range of risk factors and biomarkers (eg, haemoglobin A1c (HbA_1c_) and estimated glomerular filtration rate (eGFR)).^23 24^ The model captures the risk of the following diabetes complications: congestive heart failure, myocardial infarction, ischaemic heart disease, stroke, blindness, ulcer, amputation and renal failure. In each year, patients are at risk of dying and/or having a diabetes-related complication. The risk of events depends on patient characteristics and any history of previous events in the model. Risk factors also change over time, for example, eGFR deteriorates with age.^24^ To reflect the Pakistani context, patient profiles are based on individuals attending a diabetes clinic in Pakistan (see section on patient population for further details). A similar approach of accounting for national patient characteristics has recently been used to model diabetes in India.^27^ To our knowledge, this is the first time the UKPDS model has been adapted in this way to Pakistan. A schematic of the diabetes model is shown in online supplemental appendix figure A1.

### Depression model

**Figure 1:**
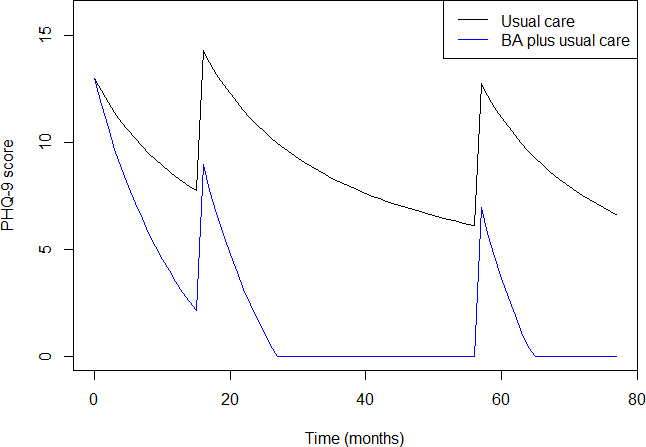
Counterfactual PHQ-9 path for a single individual with usual care (black line) or usual care plus BA (blue line). In both cases, the individual begins the model at the start of a depressive episode and has two additional episodes at month 15 and month 55. Between episodes, the individual recovers gradually, with the rate of recovery being twice as fast with BA. The PHQ-9 score at the beginning of a new episode is determined by the baseline PHQ-9 score and the PHQ-9 score before the episode began. PHQ-9 score cannot go above 27 or below 0. BA, behavioural activation; PHQ-9, Patient Health Questionnaire-9.

Depression is modelled as a cyclical disease in which patients can potentially experience multiple depressive episodes.^14 28^ Employing a novel approach to depression modelling, we modelled outcomes at the level of individual depressive symptom scores captured by Patient Health Questionnaire-9 (PHQ-9) trajectories over time (see figure 1). Individuals enter the model in a depressive episode, they then gradually recover (ie, their PHQ-9 score decreases) with the rate of recovery dependent on the time since the episode began and the treatment they receive. Throughout, they are at risk of having a new depressive episode, even if they have not recovered from the previous episode. If they experience a new episode, their PHQ-9 score increases to a value which represents episodic depression for them (this depends on their baseline PHQ-9 score and their lowest PHQ-9 score in the current episode), after which they begin recovering and the cycle starts again. Evidence to inform the initial distribution of PHQ-9, the recovery rate and the risk of a new depressive episode was taken from the INDEPENDENT study, a randomised controlled trial (RCT) of individuals with depression and diabetes in India^29^ (further details in online supplemental appendix A3).

### Interaction between diabetes and depression

**Figure 2:**
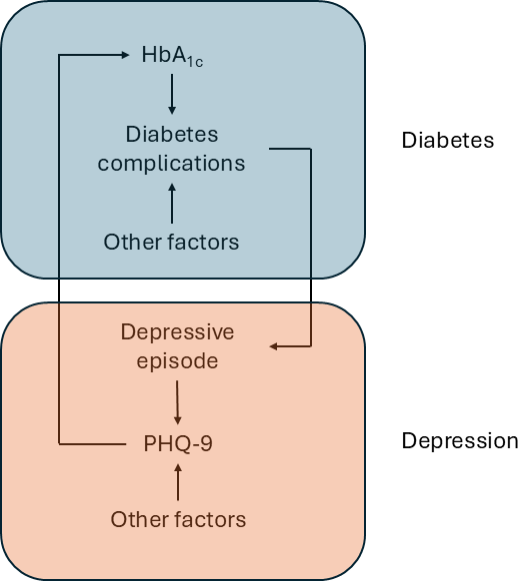
Two-way interaction between diabetes and depression. HbA1c, haemoglobin A1c, PHQ-9, Patient Health Questionnaire-9.

The model includes a two-way interaction between diabetes and depression. Depression increases the risk of future diabetes-related events by impacting on an individual’s HbA_1c_ levels, for example, because depression may result in worse self-management of diabetes.^30^ The occurrence of diabetes adverse events is increasing the risk of new depressive episodes. The relationship between PHQ-9 and future HbA_1c_ was based on the INDEPENDENT study.^29^ The increased risk of depressive episodes following diabetes-related complications was based on a published study examining the relationship between complications and incidence of depression (see online supplemental appendix A3 for further details).^31^ A schematic summarising the mechanism of this interaction is given in figure 2.

### Treatment effect for BA

A large, network meta-analysis of psychological therapies found that BA relative to ‘care as usual’ resulted in a standardised mean difference (SMD) of −0.73 with a 95% CI –0.95 to –0.52, where negative values indicate improvement on a continuous outcome scale.^32^ To transport this treatment effect into our model, we assume that the BA impacts PHQ-9 through modifying the rate of recovery during a depressive episode. A calibration approach was used to ‘back calculate’ the change in rate of recovery with BA required to result in a −0.73 SMD on PHQ-9.^33 34^ It was found that an SMD for PHQ-9 of 0.95 was consistent with a doubling of the rate of recovery with BA relative to usual care. See figure 1 for an illustration of the impact of BA on the PHQ-9 time path and see online supplemental appendix A3 for full details of the analysis, including the characterisation of uncertainty.

### Patient population

Patient profiles were built based on a representative population of individuals with diabetes from Pakistan using the Baqai Institute of Diabetology & Endocrinology (BIDE) patient registry containing 28 942 individuals.^35^ Information on disease history and heart rate not captured in the registry was imputed using external data.^14 29 36^ The BIDE register did not include information on depression, so PHQ-9 scores were estimated for individuals based on the relationship observed between covariates and PHQ-9 in the INDEPENDENT trial,^29^ with uncertainty in predictions captured. Further, missing covariates were imputed using multiple imputation by chain equations, which produces imputations accounting for the underlying uncertainty. To account for variability in patients, we randomly sampled patient profiles from the created data set. It was assumed that the joint distribution of patient characteristics in our patient profile data set (based on BIDE and other sources) represented the joint distribution in the population of interest. Full details of the development of the patient population are available in the online supplemental appendix and descriptive statistics of the patient population for the analysis are provided in online supplemental table A2.

### Costs and quality of life

#### Costs

A targeted review of Pakistani costing studies was conducted to identify costs for the model. No studies were identified which provided all the necessary costing information reflecting all of the clinical events associated with diabetes. However, a recent high-quality study looking at general management costs associated with diabetes was identified.^37^ This provided an estimate of background diabetes costs (ie, routine care for diabetes and excluding diabetic events and complications) for Pakistan. Alva *et al* provided an estimate of background diabetes costs, diabetes event and complication-related costs for the UK.^38^ To estimate costs of diabetes-related events in Pakistan, we calculated the ratio of background costs for Pakistan (using Gupta *et al*^37^) and the UK (using Alva *et al*^38^) and assumed that the ratio of costs was constant across all events to estimate Pakistan costs. Costs were inflated to 2020 USD values, see online supplemental appendix table A4 for full list of costs and for details on conversion and inflation adjustment.

The cost of care per depressive episode was estimated to be US$67.07, this includes inpatient care, outpatient care and costs of medicines.^39^ A cost per course of BA of US$15 was estimated based on the expected resource use from the pilot trial protocol, which was applied in addition to the cost of usual care in the BA intervention arm for each depressive episode.^8^

#### Disability weights

Quality of life impacts for each diabetes-related complication were captured in DALY weights.^40^ For depression, the PHQ-9 score for each individual in each month was classified into none, mild, moderate and severe. DALY weights were applied to each of these categories, and the average DALY score for each year was calculated. In calculating DALYs, the life expectancy for each age group was taken from the WHO global burden of disease 1990–2019 survey to reflect years of life lost.^41^ See online supplemental appendix table A5 for full information.

### Economic analysis

#### Cost-effectiveness analysis

An intervention is considered cost-effective if the health produced by the intervention exceeds the health which could be generated elsewhere using the same resources. Cost-effectiveness was assessed using a cost-effectiveness threshold of US$183 per DALY averted.^42^,^44^ This is an estimate of the marginal productivity for the Pakistani health system reflecting how much health could be generated elsewhere if resources were used for alternative purposes, that is, an intervention must avert a DALY for less than US$183 or it would not be considered a cost-effective use of resources. Cost-effectiveness is presented in terms of incremental cost-effectiveness ratios (ICER), incremental net health benefits and incremental net monetary benefits (INMB). Costs and outcomes were discounted at a rate of 3% per annum.^21^

#### Headroom analysis

Headroom analysis allows us to estimate the maximum price of a treatment at which it remains cost-effective for a given level of effectiveness.^16 17^ Here, it is used to estimate the maximum cost at which BA is the cost-effective option, that is, we estimated the maximum cost of BA for which the ICER of BA vs usual practice is equal to the cost-effectiveness threshold of US$183. As individuals potentially experience multiple episodes of depression, the total cost is split over the expected number of depressive episodes to calculate a maximum cost for BA per episode. Further details of the headroom analysis are provided in online supplemental appendix A6.

#### VOI analysis

VOI methods quantify the costs of uncertainty in health or monetary terms in terms of the chance and consequences of making a wrong decision (ie, incorrectly implementing a non-cost-effective treatment). These methods allow for the estimation of the value of collecting information in a trial.^18 19^ Expected value of partial perfect information (EVPPI) methods allow analysts to quantify the value of collecting information on individual outcomes or groups of outcomes. In the case of DiaDeM, we use EVPPI to compare the value of resolving uncertainty in 21 parameter groups (see online supplemental appendix table A7.1), to identify those which are potentially most important to collect additional evidence on in the DiaDeM trial. These parameters are classified as short, medium and long term, reflecting the degree to which hypothetical trials with short, medium and long-term follow-up could gather information on each parameter group. For example, a trial would require a long follow-up to provide substantial information on the risk of diabetes events and mortality. However, only a short follow-up would be required to observe the costs associated with routine depression care. Note that some aspects of the model (such as the effect of depression on diabetes) are captured by just one parameter, whereas others (such as the evolution of eGFR over time) require many parameters.

The VOI analysis is carried out assuming the headroom cost of BA to maximise uncertainty, with alternative costs of BA used for scenario analyses. To estimate the population EVPPI, we multiply the individual EVPPI estimates by an estimate of the prevalent population with diabetes and depression; this is 12.84 million in Pakistan.^45^,^47^ Full details are provided in online supplemental appendix A7.

### Generating model predictions

This is a microsimulation model which generates estimates for population outcomes by repeatedly simulating individuals and recording their outcomes. Therefore, it is necessary to check the number of patients required to achieve convergence of population-level results. The model was found to have reasonable convergence after simulating approximately 8000 individuals. To reflect uncertainty in model inputs, a probabilistic sensitivity analysis was carried out by drawing 1000 times from the sampling distribution of each input parameter.^15^ Outcomes (eg, life years, DALYs) were computed for each combination of input values resulting in a posterior distribution for each outcome. Point estimates were computed from the mean of these distributions and credible intervals by computing the relevant quantiles.

## Results

### Clinical outcomes

Table 1 summarises the clinical outcomes predicted by the model including depression outcomes, diabetes events and cause of death. As described above, BA is expected to double the rate of recovery from depressive episodes, meaning that over their lifetime, those who receive BA are predicted to avoid 3.24 years of mild depression (PHQ-9 >5) and 0.65 years of moderate depression (PHQ-9 >10) relative to usual care. BA is expected to reduce the rate of nearly all diabetes events by lowering HbA_1c_. This reduction in diabetes events increases life expectancy by 0.27 years on average. BA also slightly reduces the rate of depressive episodes through the interaction with diabetes outcomes. Lower PHQ-9 results in lower HbA,_1c_ resulting in a lower risk of diabetes events and ultimately a lower risk of a depressive episode.

**Table 1:** Clinical outcomes simulated in the DiaDeM model, covering depression outcomes, diabetes-related outcomes, mortality and cause of death

Outcome	UC	UC plus BA	UC plus BA vs UC (incremental analysis)
	Mean (80% CrI)	Mean (80% CrI)	Mean (80% CrI)
Depression outcomes			
Years PHQ-9>5 (mild depression)	4.39 (3.69 to 5.07)	1.14 (0.91 to 1.4)	−3.24 (−3.83 to –2.7)
Years PHQ-9>10 (moderate depression)	0.86 (0.72 to 1.02)	0.21 (0.18 to 0.24)	−0.65 (−0.81 to –0.51)
Number of depressive events per 1000 person years	236 (212 to 264)	234 (210 to 261)	−2 (−4 to 0)
Diabetes events per 1000 person years			
First MI	24 (11.9 to 36.8)	23 (11.4 to 35.4)	−0.9 (−1.8 to –0.2)
Second MI	5.3 (1.7 to 10.1)	5.2 (1.7 to 9.9)	−0.2 (−0.5 to 0.2)
First stroke	15.5 (3.8 to 31.5)	14.7 (3.7 to 29.9)	−0.8 (−1.7 to –0.1)
Second stroke	5.4 (0.1 to 12.4)	5.2 (0.1 to 12.4)	−0.2 (−0.7 to 0.1)
CHF	10.1 (2 to 22.1)	10.1 (2 to 22.2)	0 (−0.4 to 0.4)
IHD	10.3 (3.8 to 18.9)	10.3 (3.7 to 18.9)	0 (−0.4 to 0.3)
First amputation	11 (1.9 to 25.9)	10 (1.8 to 23)	−1 (−2.5 to –0.1)
Second amputation	4.6 (0.6 to 11.4)	4 (0.5 to 10)	−0.6 (−1.5 to 0)
Blindness	5.9 (1.1 to 12.3)	5.4 (1 to 11.4)	−0.5 (−1.1 to 0)
Renal failure	4.3 (0.1 to 12.1)	4.3 (0.2 to 11.9)	0 (−0.2 to 0.2)
Ulcer	3.8 (0.2 to 10)	3.4 (0.2 to 8.9)	−0.4 (−1.1 to 0)
Cataract	24.9 (21.5 to 28.8)	24.7 (21.4 to 28.4)	−0.3 (−0.7 to 0.1)
Severe hypo	14 (13.7 to 14.3)	14.1 (13.7 to 14.4)	0.1 (−0.4 to 0.6)
Mortality			
Life years	19.07 (14.38 to 23.81)	19.34 (14.73 to 23.87)	0.27 (0.03 to 0.52)

BA, behavioural activation; CHF, congestive heart failure; CrI, credible interval; DiaDeM, Developing and evaluating an adapted behavioural activation intervention for depression and diabetes in South Asia; IHD, ischaemic heart disease; MI, myocardial infarction; PHQ-9, Patient Health Questionnaire-9; UC, usual care.

### Cost-effectiveness analysis

Table 2 summarises the discounted results comparing usual care against usual care plus BA including and excluding the US$15 BA treatment costs. BA plus usual care results in lower overall healthcare costs and more DALYs averted than usual care. At US$15 per course, BA dominates usual care, being less costly and more effective.

**Table 2:** Summary of cost-effectiveness results comparing BA in addition to usual care versus usual care alone

Treatment option	Total healthcare costs (95% CrI)	Incremental costs, over usual care (95% CrI)	DALYs averted (95% CrI)	Incremental DALYs averted, over usual care (95% CrI)	Incremental net monetary benefit, over usual care (95% CrI)	ICER
Usual care	US$10 839 (US$7205 to US$17 088)	–	−3.5 (7.96 to −9.58)	–	–	
BA (excluding BA treatment costs)	US$10 742 (US$7187 to US$16 803)	−US$97 (−US$517 to US$142)	−2.52 (9.07 to −8.70)	0.98 (1.86 to 0.45)	US$276.63 (US$23.92 to US$717.12)	Dominates usual care
BA (including BA treatment costs of $15)	US$10 792 (US$7237 to US$16 854)	−US$47 (−US$467 to US$192)	−2.52 (9.07 to −8.70)	0.98 (1.86 to 0.45)	US$226.48 (−US$26.22 to US$666.97)	Dominates usual care

BA, behavioural activation; CrI, credible interval; DALYs, disability-adjusted life-year; ICER, incremental cost-effectiveness ratio.

### Headroom analysis

As shown in table 2, the INMB for BA excluding treatment costs was US$276.63 (US$23.92 to US$717.12). The average number of depressive episodes per individual in the BA arm discounted to present value was 3.34 (2.56 to 4.23) resulting in a headroom cost of US$82.58 for a course of BA (US$8.60 to US$214.10). This is the maximum cost per course of treatment per person which would be expected to be cost-effective. It should be noted that this is considerably higher than the estimated US$15 based on expected resource use from the DiaDeM pilot trial. Due to uncertainties in the evidence, the headroom estimate was associated with considerable uncertainty, with the 95% credible interval ranging from US$8.60 to US$214.10. This headroom estimate was used to inform the maximum number of sessions per person for the DiaDeM trial.

### VOI analysis

For the base case, VOI is calculated based on the headroom cost for the intervention US$82.58. Table 3 presents results scaled to reflect the prevalent population in Pakistan (12.84 million). EVPPI estimates the value of resolving uncertainty in a group of parameters. All else being equal (eg, evidence will cost the same to produce, will resolve the same amount of uncertainty) higher EVPPI values for a given group of parameters indicate that there is potentially more value in gathering data on this group. The largest EVPPI value is for risk of diabetes events and mortality (US$728.95m). Data on the risk of these events would require long-term follow-up and are likely to be best collected using a registry rather than as part of an RCT. Data could be captured on the PHQ-9 time path over a shorter period (say 1 year) and are expected to provide significant value (US$473.3m). There is also value in learning the BA treatment effect (US$18.16m), which should be feasible over the time period of a trial. There is expected to be significant value in understanding the trajectories of many of the parameters used in the model, the highest being for high-density lipoprotein cholesterol (US$386m). These parameter groups require medium to long-term follow-up to capture fully. However, shorter trial designs may provide partial information on these outcomes, capturing the trajectories over the trial period.

**Table 3:** EVPPI results for estimated protocol cost of BA and headroom cost

Group of parameters	EVPPI for population in millions US$ (rank)
	Headroom cost, US$82.58
Short-term parameters	
BA treatment effect	US$18.16m (15)
PHQ-9 time path with usual care	US$473.3m (2)
Effect of depression on diabetes	US$395.58m (3)
Costs of routine depression care	US$0.03m (19)
Costs of routine diabetes care	US$0m
Medium term parameters	
Time path for HbA1c	US$243.81m (10)
Time path for BMI	US$231.96m (11)
Time path for LDL cholesterol	US$354.58m (6)
Time path for systolic blood pressure	US$277.29m (9)
Time path for HDL cholesterol	US$386m (4)
Time path for haemoglobin	US$0m
Time path for white blood cell count	US$355.91m (5)
Time path for heart rate	US$169.83m (12)
Time path for smoking	US$154.04m (13)
Time path for peripheral vascular disease	US$37.12m (14)
Time path for microalbuminuria	US$321.5m (7)
Time path for atrial fibrillation	US$0.81m (18)
Time path for eGFR	US$293.04m (8)
Long-term parameters	
Effect of diabetes complications on depression	US$6.68m (16)
Costs associated with diabetes events	US$5.69m (17)
Risk of diabetes events and mortality	US$728.95m (1)
Probability of BA being cost-effective	42%

BA, behavioural activation; BMI, body mass index; eGFR, estimated glomerular filtration rate; EVPPI, expected value of partial perfect information; HbA_1c_, haemoglobin A1c; HDL, high-density lipoprotein; LDL, low-density lipoprotein; PHQ-9, Patient Health Questionnaire-9.

Online supplemental appendix A7 presents results for four further sensitivity analyses exploring the impact of different assumptions about the cost of BA: (1) US$15 based on the expected resource use from the trial intervention, (2) US$65.65 found in an Indian study with intensive compliance efforts,^48^ (3) US$8.60 and (4) US$214.10, the lower and upper credible intervals from the headroom analysis, respectively. In each case, the value of further research is lower than the base case, this is because the headroom analysis chooses the BA price which maximises uncertainty.

## Discussion

In this paper, we developed a novel MLTC model of diabetes and depression to assess the potential benefits of a BA intervention and to carry out headroom and VOI analyses with the aim of informing the DiaDeM intervention design and trial. The model developed for this paper will also be used to assess the long-term cost-effectiveness of the DiaDeM BA intervention following the culmination of the definitive trial.

From the analysis, BA is expected to result in considerably less time spent in depressive episodes (3.2 years of mild depression and 0.65 years of moderate depression avoided) and is also expected to reduce the occurrence of diabetes-related adverse events. BA was found to improve health outcomes and reduce costs. In the headroom analysis, we found that the maximum price at which BA was expected to be cost-effective was US$82.58 per course of treatment per person based on a treatment effectiveness which doubles the rate of recovery from depression (95% credible interval ranging from US$8.60 to US$214.10). Typical wage rates for the relevant staff in Pakistan are approximately US$2/hour and each session (other than the first session) is expected to take 30 min, resulting in a cost of US$1 per session (excluding preparation costs). The lower bound estimate for the headroom analysis is US$8.60, with a cost per session of US$1. This implies that the intervention is expected to be cost-effective even if requiring over eight sessions per person (US$8.6/US$1>8). The VOI analysis found that there was considerable value in collecting additional information on the different parameters in the model, including large value in short-term outcomes such as the effect of depression on diabetes (US$395.58m) and PHQ-9 time path (US$473.3m). These assessments fed into both the intervention and trial design for DiaDeM. This analysis also suggests that there is considerable value in reducing uncertainty about the time path of biomarkers such as HbA_1c_ and body mass index. This can provide an estimate of the value of setting up longitudinal data collection for each of these measures. This could then be compared against the expected value of other research projects competing for funding.^49^

This paper employed a novel approach to predicting outcomes in depression by modelling individual PHQ-9 scores over time. This can be compared with models which consider a single episode of depression^50^,^52^ and models which are based on discrete states of depression.^14 28^ This approach may be more intuitive, and by not classifying patients into broader categories, we do not lose information and can better model individual level variation in outcomes. This model structure also allowed us to use individual patient data to estimate the impact that changes in depressive symptoms (PHQ-9) have on diabetes (through HbA_1c_). External data were used to inform the impact of diabetes-related complications on rates of new depressive episodes.^31^ This approach to modelling two-way interactions uses separate data sources to estimate the independent effect of diabetes on depression and vice versa. The endogeneity between the MLTCs is then imposed by the model structure. This makes strong implicit assumptions about the underlying causal structure of disease interactions. A more sophisticated approach would require long-term longitudinal data on diabetes and depression outcomes. Careful application of causal inference methods would be required in this case to estimate the time-dependent endogenous relationship between the MLTCs.^53^,^55^

Health system costs and out-of-pocket costs are combined into healthcare costs in this analysis. A full multisector analysis would be required to treat these costs as falling on different budgets, consumption in the case of out-of-pocket costs and the health budget in the case of health system costs.^20 56^ This is potentially an important area of further research.

It should be noted that EVPPI represents the value of eliminating all uncertainty in a group of parameters. Therefore, it represents an upper bound for the value of research. For the task of approximating the relative value of collecting information on different parameters, EVPPI may be a reasonable approximation. However, comparing EVPPI does not consider the (potentially differential) rates at which uncertainty in a given group of parameters is resolved and how this interacts with trial design. An expected value of sample information analysis would be required to fully capture this aspect of research design.^15^ This was not carried out here due to computational and evidentiary challenges.^57 58^ As a pragmatic alternative, parameter groups were split into short, medium and long term to capture the impact of trial follow-up length, which is a novel approach to addressing the problem.

The analysis was carried out to inform the DiaDeM trial which will be carried out in both Pakistan and Bangladesh. We focused on Pakistan only because of lack of data on patient level and appropriate cost data for routine costs for people with diabetes or costs associated with major cardiovascular events. This is an important limitation of our pretrial analysis because of potential differences between these countries in, for example, costs, health behaviours and care delivery. Following the completion of the DiaDeM, the model will be parameterised for both Bangladesh and Pakistan based on the data collected in the trial.

Though there were more data for Pakistan than Bangladesh, there were still important data limitations for the Pakistani context. Where necessary, data from other countries were used to parameterise the model. For example, the PHQ-9 data were from an Indian study and disease history was imputed based on the relationship between risk factors estimated based on UK data. Data quality was also an issue for sources within Pakistan, for example, the cost of care per depressive episode was based on a secondary care facility which may not be representative of practice. For the UKPDS diabetes model, the risk of events is based on patient characteristics (eg, age, smoking status, HbA_1c_). These distributions of characteristics in the population were based on Pakistani data; however, the risk equations linking characteristics to outcomes were based on longitudinal UK data. These assumptions around the generalisability of the evidence to Pakistan are an important limitation of the analysis and highlight the need for more in-country research to better inform the model. The model could also be improved in future by further dialogue with local stakeholders and patients. The analysis in this paper does not consider screening or costs involved in identification; therefore, the results are implicitly based on the case in which individuals are perfectly identified (all true positives). This also relates to the choice of comparator in the model: usual care. Those who are not identified as having depression will go without any treatment. This no treatment option was not included in the analysis as it was not considered a relevant policy alternative. However, it would be necessary to include in an analysis which included screening which may be relevant for decision makers.

### Conclusions

We found that BA had the potential to be a cost-effective intervention compared with usual care for patients with both depression and diabetes in Pakistan, improving morbidity and mortality and reducing costs. Efforts must be made to keep the BA treatment cost low, and uncertainty remains over the impact of depression on diabetes and the trajectory of depression. This evidence has helped to inform the design of the DiaDeM intervention by highlighting the value of collecting evidence on the impact of depression on diabetes and the time path of PHQ-9 scores. The model developed will be used to estimate the cost-effectiveness of the intervention following completion of the definitive trial.

## Supplementary material

10.1136/bmjopen-2024-092158 online supplemental file 1

10.1136/bmjopen-2024-092158 online supplemental file 2

## Data Availability

Data may be obtained from a third party and are not publicly available.
