# Supplementary material for: Modelling diabetes and depression in Pakistan: using economic modelling to inform intervention design and a clinical trial of a behavioural activation intervention
Source: BMJ Open. 2025 May 19;15(5):e092158. doi: 10.1136/bmjopen-2024-092158 (PMC12090864; doi:10.1136/bmjopen-2024-092158)
Supplement: online supplemental file 1 [file bmjopen-15-5-s001.docx]

Appendix

A1: Diabetes model structure

[INSERT FIG A1]

A2: Patient population

Patient profiles were built from the Baqai Institute of Diabetology & Endocrinology (BIDE) patient registry containing 28,942 individuals (1). Table A2 summarises the patient information that was captured in BIDE.

**Imputing missing observations in BIDE**

Note that the missingness in some of the biomedical variables was high (e.g. albuminuria 98% missing, white blood cell count 81% missing). To maintain the representativeness of the data we imputed the missing information using Multivariate Imputation by Chained Equations (MICE) which maintains correlations between inputs (2, 3). Predictive mean matching was used for numeric variables and logistic regression was used for binary variables. Due to the large size of BIDE, 5 imputation datasets were generated to reflect variability in predictions for each individual. This number was chosen to limit the size of the data set required for the modelling so as to ensure efficient calculation. Sampling with replacement was used to construct the final set of patient profiles. Patient profiles were restricted to be 18 years or above to match the population of interest in the DiaDeM trial (10 patients excluded from the dataset). Due to challenges with administrative data, we have assumed implausible values as missing and imputed them. For example, we considered it implausible for the same individual to be below 1.2 meters while being above 140kg in weight. This only impacts a small number of observations and should have minimal impact on results.

**Predicting covariates not included in BIDE**

As shown in Table A2, BIDE included data on 14 out of 28 patient characteristics required for the model. Other sources were used to inform the covariates which were absent from BIDE.

- Following Kearns et al, absent information on disease history were predicted using a logistic model based on the 2013 national diabetes audit in the UK (4, 5).
- Heart rate, atrial fibrillation, peripheral vascular disease and history of hypoglycaemic events were predicted based on models estimated from the INDEPENDENT trial (6). Covariate choice was based on goodness of fit as measured by Akaike information criterion (AIC). Where sufficient numbers of observations were available backwards selection was used, otherwise the best single predictor was chosen.
- History of cataracts was imputed by simulating age at cataract surgery from a Pakistani study and comparing this to the age at baseline (7).
- PHQ-9 data were also not available for the patients in BIDE. To maintain correlations between PHQ-9 and diabetic characteristics, baseline PHQ-9 was predicted based on ordinary least squares (OLS) models estimated from the INDEPENDENT trial (6). Covariate choice was again based on AIC and backwards selection. To reflect the DiaDeM population, only patients with a predicted PHQ-9 of 5 or above were included in the analysis. To avoid non-sensical scores, any predictions above the range of the instrument were set to the maximum value, PHQ-9 of 27.

*Table A2: Summary of simulated patient profiles used in the model. SD = standard deviation, PHQ-9 = patient health questionnaire-9; HbA1c = haemoglobin A1c; BMI = body mass index; LDL = how density lipoprotein; HDL = high density lipoprotein, Hg = haemoglobin*

| **Patient characteristics** | **Value** | **Details of predictive model** | **Source** |
| --- | --- | --- | --- |
| Age at diagnosis (years) | Mean = 44.15  SD = 10.56  95% range = 25.34, 66.11  Missing = 0% | - | BIDE |
| Years of diabetes at presentation | Mean = 9.41  SD = 7.79  95% range = 0.18, 28.51  Missing = 0% | - | BIDE |
| Female | Mean = 46.47%  Missing = 0% | - | BIDE |
| South Asian ethnicity | Mean = 100% | - | Assumption |
| HbA_1c_ (%) | Mean = 9.6  SD =2.34  95% range = 6, 14.5  Missing = 48% | - | BIDE |
| Systolic blood pressure (mm Hg) | Mean = 128.44  SD = 18.26  95% range = 100, 170  Missing = 0% | - | BIDE |
| LDL cholesterol (mmol/l) | Mean = 275.75  SD = 103.87  95% range =100.85, 499.1  Missing = 57% | - | BIDE |
| HDL cholesterol (mmol/l) | Mean = 90.97  SD = 24.52  95% range = 46.55, 144.82  Missing = 63% | - | BIDE |
| BMI | Mean = 28.72  SD = 5.47  95% range =19.9, 40.58  Missing = 0% | - | BIDE |
| Albuminuria | Mean = 27.06%  Missing = 98% | - | BIDE |
| Heart rate (beats per minute) | Mean = 85.67  SD = 11.48  95% range = 62.96, 107.76 | Linear regression with covariates: sex, HbA_1c_ and eGFR | Ali et al. 2020 |
| White blood cell count (1x10^6/ml) | Mean = 9.33  SD = 4.11  95% range = 5, 18.1  Missing = 81% | - | BIDE |
| Haemoglobin (g/dl) | Mean = 12.48  SD = 2.2  95% range = 8.1, 16.6  Missing = 86% | - | BIDE |
| Estimated glomerular filter rate (ml/min/1.73m^2) | Mean = 64.56  SD = 20.06  95% range = 21, 103  Missing = 87% | - | BIDE |
| Atrial fibrillation | Mean = 1.25% | Logistic regression with covariates: HbA_1c_ | Ali et al. 2020 |
| Peripheral vascular disease | Mean = 1.62% | Logistic regression with covariates: sex | Ali et al. 2020 |
| Current smoking | Mean = 9%  Missing = 0% | - | BIDE |
| History of congestive heart failure | Mean = 0.32% | Logistic regression with covariates: age, sex, ethnicity and BMI | 2013 national diabetes audit |
| History of myocardial infarction | Mean = 1.65% | Logistic regression with covariates: age, sex, ethnicity and BMI | 2013 national diabetes audit |
| History of ischaemic heart disease | Mean = 1.07% | Logistic regression with covariates: age, sex, ethnicity and BMI | 2013 national diabetes audit |
| History of stroke | Mean = 0.35% | Logistic regression with covariates: age, sex, ethnicity and BMI | 2013 national diabetes audit |
| History of blindness | Mean = 0.01% | Logistic regression with covariates: age, sex, ethnicity and BMI | 2013 national diabetes audit |
| History of ulcer | Mean = 7.53%  Missing = 0% | - | BIDE |
| History of amputation | Mean = 0.06% | Logistic regression with covariates: age, sex, ethnicity and BMI | 2013 national diabetes audit |
| History of renal failure | Mean = 0.09% | Logistic regression with covariates: age, sex, ethnicity and BMI | 2013 national diabetes audit |
| History of cataracts | Mean = 36.49% | Simulated age at cataract surgery and compare to age at baseline | Bourne et al. 2007 |
| History of hypoglycaemic events | Mean = 2.25% | Logistic regression with covariates: sex and LDL | Ali et al. 2020 |
| PHQ-9 | Mean = 12.16  SD = 2.64  95% range = 7, 17 | Linear regression with covariates: age, age squared, sex, BMI and eGFR | Ali et al. 2020 |

A3: Depression modelling

The initial distribution of PHQ-9 was assumed to represent patients experiencing a depressive episode (see Table A2). In every cycle that a patient does not have a new depressive episode, those allocated to routine care are assumed to recover at a rate which was calculated from the INDEPENDENT trial (6). Patients were assumed to be episodic at the start of the INDEPENDENT trial. A generalised linear model (GLM) with an identity link, gamma distribution and covariate for time, was fitted to capture recovery from this initial episode. This allowed for the rate of recovery to depend on time since the episode. Each individual was assumed to recover at the same rate after each subsequent episode. Treatment with BA was assumed to alter this rate of recovery (see “Calibration for treatment effect of BA” for further details).

Patients were classified as having a new depressive episode if their PHQ-9 score increased by 5 points between any two time points in the INDEPENDENT trial. A five-point cut off was chosen as PHQ-9 disease classification cut-offs are based on five-point changes (8). When estimating the rate of recovery patients were censored if they experienced a new depressive episode, n = 123 (30.4%). To model the rate of new depressive episodes, a range of parametric survival models were fitted to the data (exponential, Weibull, Gompertz, log normal, log logistic and generalised gamma) with the starting point being the time of the previous depressive episode. Log normal was found to fit the data best according to AIC and so was used in the base case. Including covariates was found not to improve data fit. The PHQ-9 score associated with a new depressive episode was estimated using an ordinary least squares (OLS) regression. It was found that PHQ-9 at the start of a new episode depended on the minimum PHQ-9 score prior to the episode and the PHQ-9 score at baseline (assumed time of initial depressive episode). The results from the models estimated are reported in Table A3.

Modelling the impact of depression on diabetes

To capture the impact of depression on diabetes, we estimated the relationship between past PHQ-9 and future HbA_1c_. We used INDEPENDENT data at baseline, 6, 12, 18 and 24 months to estimate the relationship between PHQ-9 (time t) and HbA_1c_ 6 months later (time t+1) (6). After controlling for the time trend in HbA_1c_ we found a statistically significant positive relationship between the outcomes (see Table A2). As HbA_1c_ is modelled using a yearly cycle, we allowed HbA_1c_ in time t to depend equally on PHQ-9 at t - 6 months and t - 12 months.

Modelling the impact of diabetes on depression

We allow diabetes complications in time *t* to increase the risk of a new depressive episode in *t + 1* (4). This relationship was estimated in a Dutch study using a logistic regression and cross-sectional data (9). After controlling for a set of relevant covariates the odds ratio for depression (PHQ-9 ≥10) was found to be 2.67 (95% confidence interval from 1.26 to 5.63) for those with two or more complications (retinopathy, nephropathy, neuropathy, myocardial infarction, stable or unstable angina pectoris and peripheral or cerebral arterial disease). The odds ratio, increasing the risk of new depressive episode, was applied every year for individuals with two or more of the complications identified in the paper.

Calibration for treatment effect of BA

As described in the body of the text, to transport the NMA relative effects into our model we must translate them into a form which can be included as relative effects into our model. We assumed that the relative treatment effect in our model would operate through allowing a different rate of recovery with BA compared to usual care. The SMD point estimate for BA vs care as usual was reported as –0.73 (95% from –0.95 to –0.52) in the NMA (which represents an improvement). This was assumed to refer to the SMD at 3 months (from correspondence with authors). Using our depression model, we could repeatedly simulate PHQ-9 time paths with different rates of recovery and estimate the SMD at 3 months compared to usual care. We used a calibration approach to find the rate of recovery which would correspond to a SMD of -0.73. This search was carried out using the optim function in R (R Core Team 2021). It was found that if recovery was twice as fast (i.e. a multiplier of 2.07) with BA compared to usual care then this would result in the target SMD (10, 11).

The above approach maps only the SMD point estimate to an increase in the BA rate of recovery compared to usual care. To reflect uncertainty relative rate of recovery with BA, we reflect the uncertainty in the SMD (95% confidence interval from –0.95 to –0.52). Assuming that the SMD and the prior for the treatment effect parameter are normally distributed we can calculate the relative rate of recovery associated with the upper (UI) and lower 95% interval (LI), then use this to estimate a standard error of 0.19 (SE = abs(UI - LI)/(2*1.96)). This results in a rate of recovery multiplier with mean 2.07 and 95% interval from 1.7 to 2.44.

*Table A3: Inputs used in predicting depression scores over time. SE = standard error, OLS = ordinary least squares, GLM = generalised linear model, PHQ-9 = patient health questionnaire-9, HbA1c = haemoglobin A1c, BA = behavioural activation.*

| **Input** | **Functional form** | **Value** | **Source** |
| --- | --- | --- | --- |
| Rate of recovery in PHQ-9 points per month, following a depressive episode with usual care | GLM with identity link, gamma family and covariate for time (month) | Time dependent:  Intercept: mean = 0.077, SE = 0.001  Month: mean = 0.004, SE = 0.0002 | Ali et al. 2020 |
| Probability of new depressive episode | Log normal survival model | Time varying, 1% per month on average  Log constant: mean = 7.32, SE = 0.067  Log sigma: mean = -0.22, SE = 0.072 | Ali et al. 2020 |
| PHQ-9 score with new depressive episode | OLS with coefficients for minimum PHQ-9 value and PHQ-9 at baseline | 4.1 + 0.95*minimum PHQ-9 value + 0.22*PHQ-9 at baseline | Ali et al. 2020 |
| Increased risk of new depressive episode with two or more diabetes complications | Logistic regression | Odds ratio: 2.67 (95% confidence interval from 1.26 to 5.63) | van Steenbergen-Weijenburg et al. 2011 |
| Impact of past PHQ-9 on present HbA_1c_ | OLS with change in HbA_1c_ between t and t-6 months as dependent variable and PHQ-9 at t-6 months as independent variable | Coefficient for lagged PHQ-9; Mean = 0.047, SE = 0.007 | Ali et al. 2020 |
| Difference in rate of recovery with BA compared to usual care | Multiplier applied to usual care rate of recovery | Mean = 2.07, SE = 0.19 | Cuijpers et al. 2021 |

A4: Costs of diabetes and depression care

The table below shows the diabetes and depression costs used in the model. All cost inputs are for the Pakistani health system and are denominated in 2020 $USD.

The background diabetes care costs for Pakistan came from Gupta et al 2020 which reported results for 2017, these were converted to Pakistani rupee (PKR), inflated to the year 2020 and then converted to USD using World Bank data (12, 13). Costs of other adverse events related to diabetes were not available in Pakistan. Therefore, for other diabetes these costs, UK costs based on Alva et al 2015 were converted to costs to Pakistani 2020 $USD based on the relationship between the Gupta et al costs and Alva et al costs for background diabetes costs (a ratio of 0.3018 was used).

*Table A4:* *Costs of diabetes and depression care. SE = standard error, BA = behavioural activation, MI = myocardial infarction, IHD = ischaemic heart disease.*

| **Input** | **Value, all costs in 2020 $USD** | **Source** |
| --- | --- | --- |
| Ratio of Pakistan costs to Alva et al 2015 | 0.3018 | Gupta et al 2020  Alva et al 2015 |
| Background diabetes costs | $299 | Gupta et al 2020 |
| Myocardial infarction | $2,216 | Alva et al 2015 |
| Ischaemic heart disease | $3,209 | Alva et al 2015 |
| Stroke | $2,383 | Alva et al 2015 |
| Heart Failure | $1,259 | Alva et al 2015 |
| Amputation | $3,696 | Alva et al 2015 |
| Blindness | $409 | Alva et al 2015 |
| Fatal myocardial infarction | $459 | Alva et al 2015 |
| Fatal ischaemic heart disease | $1,137 | Alva et al 2015 |
| Fatal stroke | $1,193 | Alva et al 2015 |
| History of MI | $551 | Alva et al 2015 |
| History of IHD | $564 | Alva et al 2015 |
| History of Stroke | $568 | Alva et al 2015 |
| History of Heart failure | $738 | Alva et al 2015 |
| History of Amputation | $1,027 | Alva et al 2015 |
| History of Blindness | $359 | Alva et al 2015 |
| Cataract | $211 | Kearns et al 2017 |
| Cost for standard depression care per episode | $67 (SE = $4.7) | Malik and Kahn 2016 |
| Cost of BA, above the costs of standard care | $15 | DiaDeM protocol (14) |

Note that costs in Alva et al 2015 are composed of inpatient and outpatient costs, with the probability of outpatient costs determined by a logistic regression. Therefore, standard errors could not be quoted for these aggregated inputs. However, the component inpatient, outpatient and logistic regression models were all estimated with using generalised linear models and so standard errors were computed for each parameter. The uncertainty in these models was reflected in the probabilistic sensitivity analysis (PSA), see section “Generating model predictions” in the main text.

A5: DALY weights for diabetes and depression

*Table A5: DALY weights for diabetes and depression. DALY = disability adjusted life year, MI = myocardial infarction, IHD = ischaemic heart disease, CHF = congestive heart failure, Amp = amputation.*

| **Event in model** | **DALY weight category from Salomon et al 2015** | **Proportion in each category** | **Source for proportion** |
| --- | --- | --- | --- |
| 1st CHF | Heart failure   - Mild 0·041 (0·026–0·062) - Moderate 0·072 (0·047–0·103) - Severe 0·179 (0·122–0·251) | 29%  19%  51% | (15) |
| 1^st^ IHD | Angina pectoris   - Mild 0·033 (0·020–0·052) - Moderate 0·080 (0·052–0·113) - Severe 0·167 (0·110–0·240) | 27%  19%  54% | (16) |
| 1^st^ MI male  1^st^ MI female  2^nd^ MI | Acute myocardial infarction   - Days 1–2 0·432 (0·288–0·579) - Days 3–28 0·074 (0·049–0·105) | Weight of 0.432 for the first 2 days, and then a weight of 0.074 for the next 26 days. | Assumption |
| 1^st^ Stroke  2^nd^ Stroke | Stroke   - Long-term consequences, mild 0·019 (0·010–0·032) - Long-term consequences, moderate 0·070 (0·046–0·099) - Long-term consequences, moderate, plus cognition problems 0·316 (0·206–0·437) - Long-term consequences severe 0·552 (0·377–0·707) - Long-term consequences, severe, plus cognition problems 0·588 (0·411–0·744) | For 1st and 2nd stroke assumed 20% in each category. | Assumption |
| Blindness | Distance vision   - Blindness 0·187 (0·124–0·260) | - | NA |
| Ulcer | Diabetes and digestive and genitourinary disease   - Diabetic foot 0·020 (0·010–0·034) | - | NA |
| 1^st^ Amp  2^nd^ Amp | Amputation   - One leg: long term, with treatment 0·039 (0·023–0·059) - One leg: long term, without treatment 0·173 (0·118–0·240) - Both legs: long term, with treatment 0·088 (0·057–0·124) - Both legs: long term, without treatment 0·443 (0·297–0·589) | For 1^st^ and 2^nd^ amputation assumed 50% treated vs not treated. | Assumption |
| Renal failure | Diabetes and digestive and genitourinary disease   - Chronic kidney disease (stage 4) 0·104 (0·070–0·147) | - | NA |
| Cataract | Distance vision   - Monocular impairment 0·017 (0·009–0·029) | - | NA |
| Depression | Major depressive disorder   - Mild episode 0·145 (0·099–0·209) - Moderate episode 0·396 (0·267–0·531) - Severe episode 0·658 (0·477–0·807) | - | NA |

A6: Headroom analysis

For the headroom analysis we first ran the model for both BA and usual care without including any incremental costs of BA (over and above usual care costs). Second, the expected incremental net monetary benefit (INMB) of the BA option was calculated. This is the gain in overall health from BA expressed in monetary terms:

INMB = ΔDALY*k – ΔTotalCost

Where ΔDALY is the additional DALYs averted with BA, *k* is the marginal productivity of the Pakistani health system and ΔTotalCost are the additional costs with BA (excluding BA specific treatment costs). Headroom analysis involves finding the cost of the BA intervention (Cost^BA^) at which INMB = 0. This is given by:

INMB - Episodes^BA^ * Cost^BA^ = 0

Cost^BA^ = INMB/ Episodes^BA^

In the model patients with repeated depressive episodes are assumed to receive ongoing treatment with the treatment they were initially allocated to. Therefore, Episodes^BA^ represents the number of depressive episodes in the BA arm discounted to present value to adjust for the timing of these costs. We estimated INMB and Episodes^BA^ using the model. To propagate uncertainty in the input parameters we repeated this for 1000 PSA samples.

A7: Value of information analysis

VOI calculates this probability of making a “wrong” decision (i.e. recommending something which is not actually cost-effective) in addition to the health consequences associated with “wrong” decisions. Jointly this allows the quantification of the maximum value of gathering further evidence to remove uncertainty and can be used to guide research decisions. Different components of a model will be associated with different degrees of uncertainty and some may be more important in the final uncertainty in whether BA is cost-effective. Expected value of partial perfect information (EVPPI) methods allow analysts to quantify the influence of single parameters or groups of parameters (17-19). In the case of DiaDeM, we use EVPPI to compare the value of resolving uncertainty in 21 parameter groups, to identify those which are potentially most important to consider collecting additional evidence on in the DiaDeM trial. The groups investigated are listed below.

*Table A7.1: Parameters and groups of parameters assessed in the value of information analysis. All parameters are grouped by the trial duration required to provide substantial observation. HbA1c = hemoglobin A1c, LDL = low density lipoprotein, HDL = high density lipoprotein, PHQ-9 = Patient Health Questionnaire-9.*

| **Follow up required** | **Name of parameter / parameter group** | **Number of parameters** |
| --- | --- | --- |
| Short term | - BA treatment effect  - PHQ-9 time path with usual care  - Effect of depression on diabetes  - Costs associated with routine depression care  - Costs associated with routine diabetes care | 1  7  1  1  1 |
| Medium term | Equations which describe evolution over time for the following risk factors:  - HbA1c  - Microalbuminuria  - Peripheral vascular disease  - Atrial fibrillation  - Smoking  - Estimated glomerular filtration rate  - Systolic blood pressure  - LDL cholesterol  - HDL cholesterol  - Body mass index  - Heart rate  - White blood cell count  - Haemoglobin | 7  9  9  4  6  26  6  7  5  7  5  6  5 |
| Long term | - Costs associated with diabetes events  - Risk of diabetes events and mortality  - Effect of diabetes complications on depression | 52  153  1 |

We used Monte Carlo with 8000 inner loops and 1000 outer loops to estimate EVPPI (17). To estimate the population EVPPI, we multiply the individual EVPPI estimates by an estimate of the prevalent population with diabetes and depression in Pakistan. The population in 2021 was 225.2 million (20), the prevalence of depression in Pakistan was estimated to be 14.62% (21) and the prevalence of depression in type two diabetes was estimated as 39% (pooling data on Bangladesh, India, and Pakistan) (22). Combining these figures gives a prevalence estimate of 12.84 million with comorbid depression and diabetes in Pakistan.

The table below provides results across a range of options for the cost of BA.

*T**able A7.2: Value of information sensitivity analysis. EVPPI = expected value of partial perfect information, USD = United States Dollar; BA = Behavioural activation; PHQ-9 = Patient Health Questionnaire-9; HbA1c = Hemoglobin A1c; BMI = Body mass index; LDL = Low density lipoprotein; HDL = High density lipoprotein; eGFR = estimated glomerular filtration rate5*

| Group of parameters | EVPPI for population in millions USD [rank] | | | |
| --- | --- | --- | --- | --- |
|  | Estimated cost of DiaDeM intervention based on protocol, $15 per person | Headroom lower credible interval, $8.60 per person | Patel et al, 2007 $65.65 per person | Headroom upper credible interval, $214.10 per person |
| Short term parameters |  |  |  |  |
| BA treatment effect | $0m | $0m | $0m | $0m |
| PHQ-9 time path with usual care | $0.18m [8] | $0m | $171.34m [5] | $0m |
| Effect of depression on diabetes | $0m | $0m | $139.69m [7] | $0m |
| Costs of routine depression care | $0m | $0m | $0m | $0m |
| Costs of routine diabetes care | $0m | $0m | $0m | $0m |
| Medium term parameters |  |  |  |  |
| Time path for HbA_1c_ | $0.89m [4] | $0.17m [4] | $85.82m [11] | $0m |
| Time path for BMI | $0m | $0m | $44.67m [13] | $0m |
| Time path for LDL cholesterol | $0.7m [5] | $0m | $127.88m [8] | $0m |
| Time path for systolic blood pressure | $0m | $0m | $94.06m [10] | $0m |
| Time path for HDL cholesterol | $0.3m [6] | $0m | $120.74m [9] | $0m |
| Time path for haemoglobin | $0m | $0m | $0m | $0m |
| Time path for white blood cell count | $1.36m [3] | $0.28m [3] | $141.22m [6] | $0m |
| Time path for heart rate | $0.22m [7] | $0m | $339.91m [3] | $0m |
| Time path for smoking | $3.25m [2] | $0.6m [2] | $419.21m [1] | $0m |
| Time path for peripheral vascular disease | $0m | $0m | $4.18m [14] | $0m |
| Time path for microalbuminuria | $0m | $0m | $197.54m [4] | $0m |
| Time path for atrial fibrillation | $0m | $0m | $0m | $0m |
| Time path for eGFR | $0m | $0m | $53.01m [12] | $0m |
| Long term parameters |  |  |  |  |
| Effect of diabetes complications on depression | $0m | $0m | $1.25m [15] | $0m |
| Costs associated with diabetes events | $0m | $0m | $0m | $0m |
| Risk of diabetes events and mortality | $14.89m [1] | $9.41m [1] | $379.55m [2] | $64.42m [1] |
| Probability of BA being cost effective | 96% | 97% | 59% | 3% |

# Appendix references

1. Baqai Institute of Diabetology & Endocrinology. Patient registry 2023 [Available from: <https://bide.edu.pk/>.

2. Royston P. Multiple Imputation of Missing Values. The Stata Journal. 2004;4(3):227-41.

3. Groothuis-Oudshoorn SvBaK. MICE: Multivariate Imputation by Chained Equations in R. Journal of Statistical Software; 2011.

4. Kearns B, Rafia R, Leaviss J, Preston L, Brazier JE, Palmer S, et al. The cost-effectiveness of changes to the care pathway used to identify depression and provide treatment amongst people with diabetes in England: a model-based economic evaluation. BMC Health Serv Res. 2017;17(1):78.

5. Health and Social Care Information Centre. National Diabetes Audit 2013 [Available from: <http://www.hscic.gov.uk/nda>.

6. Ali MK, Chwastiak L, Poongothai S, Emmert-Fees KMF, Patel SA, Anjana RM, et al. Effect of a Collaborative Care Model on Depressive Symptoms and Glycated Hemoglobin, Blood Pressure, and Serum Cholesterol Among Patients With Depression and Diabetes in India: The INDEPENDENT Randomized Clinical Trial. JAMA. 2020;324(7):651-62.

7. Bourne R, Dineen B, Jadoon Z, Lee PS, Khan A, Johnson GJ, et al. Outcomes of cataract surgery in Pakistan: results from the Pakistan National Blindness and Visual Impairment Survey. British journal of ophthalmology. 2007;91(4):420-6.

8. Kroenke K, Spitzer RL, Williams JB. The PHQ-9: validity of a brief depression severity measure. J Gen Intern Med. 2001;16(9):606-13.

9. van Steenbergen-Weijenburg KM, van Puffelen AL, Horn EK, Nuyen J, van Dam PS, van Benthem TB, et al. More co-morbid depression in patients with Type 2 diabetes with multiple complications. An observational study at a specialized outpatient clinic. Diabet Med. 2011;28(1):86-9.

10. Kennedy MC, O'Hagan A. Bayesian calibration of computer models. Journal of the Royal Statistical Society: Series B (Statistical Methodology). 2001;63(3):425-64.

11. Alarid-Escudero F, MacLehose RF, Peralta Y, Kuntz KM, Enns EA. Nonidentifiability in model calibration and implications for medical decision making. Medical Decision Making. 2018;38(7):810-21.

12. World Bank. Consumer Price Index: Pakistan 2022 [Available from: <https://data.worldbank.org/indicator/FP.CPI.TOTL?locations=PK>.

13. World Bank. Official exchange rate vs USD 2022 [Available from: <https://data.worldbank.org/indicator/PA.NUS.FCRF?locations=PK>.

14. ISRCTN registry. ISRCTN40885204. Randomized controlled trial of DiaDeM, an adapted behavioural activation intervention, for people with depression and diabetes in South Asia: BMC; 2023 [Available from: <https://www.isrctn.com/ISRCTN40885204>.

15. Lippi G, Sanchis-Gomar F. Global epidemiology and future trends of heart failure. AME Med J. 2020;5(15):1-6.

16. Moran AE, Forouzanfar MH, Roth GA, Mensah GA, Ezzati M, Flaxman A, et al. The global burden of ischemic heart disease in 1990 and 2010: the Global Burden of Disease 2010 study. Circulation. 2014;129(14):1493-501.

17. Briggs A, Sculpher M, Claxton K. Decision modelling for health economic evaluation: Oup Oxford; 2006.

18. Fenwick E, Steuten L, Knies S, Ghabri S, Basu A, Murray JF, et al. Value of Information Analysis for Research Decisions-An Introduction: Report 1 of the ISPOR Value of Information Analysis Emerging Good Practices Task Force. Value Health. 2020;23(2):139-50.

19. Rothery C, Strong M, Koffijberg HE, Basu A, Ghabri S, Knies S, et al. Value of Information Analytical Methods: Report 2 of the ISPOR Value of Information Analysis Emerging Good Practices Task Force. Value Health. 2020;23(3):277-86.

20. World Bank. Population, total for Pakistan 2020 [Available from: <https://data.worldbank.org/country/pakistan>.

21. Akhtar S, Nasir JA, Abbas T, Sarwar A. Diabetes in Pakistan: A systematic review and meta-analysis. Pak J Med Sci. 2019;35(4):1173-8.

22. Uphoff, Newbould L, Walker I, Ashraf N, Chaturvedi S, Kandasamy A, et al. A systematic review and meta-analysis of the prevalence of common mental disorders in people with non-communicable diseases in Bangladesh, India, and Pakistan. J Glob Health. 2019;9(2):020417.
