# Supplementary material for: Modelling diabetes and depression in Pakistan: using economic modelling to inform intervention design and a clinical trial of a behavioural activation intervention
Source: BMJ Open. 2025 May 19;15(5):e092158. doi: 10.1136/bmjopen-2024-092158 (PMC12090864; doi:10.1136/bmjopen-2024-092158)
Supplement: Supplementary file 3 [file bmjopen-2024-092158.reviewer_comments.pdf]

PEER REVIEW HISTORY

BMJ Open publishes all reviews undertaken for accepted manuscripts. Reviewers are asked to complete a checklist review form and are provided with free text boxes to elaborate on their assessment. These free text comments are reproduced below.

ARTICLE DETAILS

Title (Provisional)

Modelling diabetes and depression in Pakistan: Using economic modelling to inform intervention design and a clinical trial of a behavioural activation intervention

Authors

Glynn, David; Saramago, Pedro; Ahmed, Naveed; Afaq, Saima; Aslam, Faiza; Basit, Abdul; Ekers, David; Fawwad, Asher; Gibbs, Naomi; Fottrell, Edward; Holt, Richard Ian Gregory; Jacobs, Rowena; Niazi, Asima Khan; Ul-Haq, Zia; Zavala, Gerardo A.; Siddiqi, Najma; Walker, Simon

---

VERSION 1 - REVIEW

**Reviewer**	**1**
**Name**	**Bhattarai, Nawaraj**
**Affiliation**	**Institute of Health & Society, Newcastle University**
**Date**	**26-Sep-2024**
**COI**	**None**

This is a novel piece of interesting work carried out by the researchers in a setting limited by the data and could pave way for further research in this area. I appreciate the methodological approach undertaken and would be nice to see this published. However, I raise few issues that I would like the authors to address:

1. The authors report that the findings of this study will be used to inform the design of the DiaDeM intervention and trial being conducted in Pakistan and Bangladesh. However, their analyses is reported to focus only in Pakistan, because of data unavailability in Bangladesh. Though these countries may be geographically and culturally similar, there might be differences in diabetic and depression profiles. Could the authors highlight what data was unavailable in Bangladesh that stopped them considering it in this study?

2. The discussion section is well written. However, considering the assumptions in the data, and some of the computations (for eg. disease history and heart rate data sourced externally and imputed, the ratio of diabetic related costs in the UK and Bangladesh (what was the ratio used?) or the PHQ-9 data from another trial- were these populations representative of the diabetic/depressive population studied here?), it would be great to see the authors

discussing any limitations arising. Also, considering the UK costs in GBP (Though, I have not checked the study referred here) and the costs reported in here in USD, how was the conversion done here? It is reported that costs were inflated to 2020 USD. Could the authors also provide the reference to the inflation indices used or report them somewhere in the supplementary file? Were there no limitations in the cost of care per depressive episode reported from a published source?

3.Cost section, Page 6 line 9. Could you please add a reference to the pilot protocol.

4. "see Error! Reference source not found" is found in multiple places in the manuscript, probably to do the hyperlinks used. Could you please correct these? Also, the manuscript is not correctly page numbered.

5. In the discussion section, page 12, line 22, where the text reads " In contrast to other models......" could you please provide a reference to these models?

6. Patient population section, page 5, line 40....the sentence "information on disease history...." starts with lower case.

**Reviewer**	**2**
**Name**	**Pokhrel, Subhash**
**Affiliation**	**Brunel University London, Health Economics Research Group**
**Date**	**04-Nov-2024**
**COI**	**None**

This manuscript describes an economic model to predict costs and benefits, primarily to inform intervention design and a clinical trial of a behavioural activation intervention to address a multiple long-term condition (diabetes-depression) in Pakistan. Whilst this is an important area of research, more work is needed to improve its quality for it to get a recommendation for publication.

Most of the following comments are around clarity, thus requiring authors to significantly rewrite and add justifications.

Major comments:

1. Co-morbidity or multi-morbidity or multiple long-term conditions (MLTCs)- more research on the evolution of these terms, how they are defined and in which context, needs to be done to choose the right term for diabetes-depression context in the manuscript. Both co-morbidity and multi-morbidity are used interchangeably whilst no discussion around multiple-long term conditions or MLTCs, to which the most recent academic debate is oriented. If it is a co-morbidity model, it is important to identify which one the primary

diagnosis was for modelling purposes. If it was a multi-morbidity or MLTC model, see point 2 below.

2. Linked to point 1 is the lack of clarity in modelling strategy. It appears that an endogenous construct is allowed (a state of both diabetes and depression is jointly determined) whilst model operationalisation used empirical values of independent impact of one on the other and vice versa (joint impact not determined within the model). Was this the limitation due to availability of data or a significant miss? There is no discussion and empirical tests conducted to establish the robustness/validity of this modelling component (a simple sensitivity analysis might not pick this up; you might need to let the model estimate the joint impact).

3. One of the major issues of this manuscript is that it imagines the readers already know everything about multi-morbidity and economic modelling, as it makes numerous assumptions and choices without critical justifications. The UK diabetes model is assumed to work with some adjustments and additional depression modules – we are not told with critical evaluation why that would be the case. Same with other approaches taken - the fact that one single study in India had similar approach does not sufficiently justify this is ok. A thorough discussion about the choice of UK model and what specific attributes make it transferable to Pakistani context is needed. An all-rounded discussion around relevance, applicability and generalisability to answer two questions really: (a) is the way UK model is structured and populated with available input data equally resonate with Pakistani context (in whose opinion)?; and (b) would the same level of the performance of the economic model be expected if implemented for Pakistani contexts?

4. Now the first of the two questions could have been answered through PPI work. It is important to clarify why PPI were not involved at all in the design, conduct, reporting and dissemination of this work. Why is this work still useful for local stakeholders including researchers and end-users of the research findings?

5. It is not clear how the EVPPI analysis would add value pragmatically? Either a clear discussion is provided with current EVPPI data or this analysis be presented in a more pragmatic way to make the findings practically useful for Pakistani decisionmakers.

6. This manuscript is written in a style that is very hard to understand, even for health economists. Consider rewriting several sentences throughout. In addition, only the last paragraph of the discussion section acknowledges some limitations although there are several.

Specific comments:

1. Abstract – co-morbidity and multi-morbidity are used interchangeably. “BA+usual care” vs usual care, rather than BA versus usual care. State key HE parameters: perspectives, time horizon, discount rate, etc. Expand abbreviations while used for the first time. Correct typo. Consider rewriting conclusion: focus on how exactly the work informed the design of

DiaDeM intervention and the definitive trial (which aspect of the findings directly led to that?).

2. Key messages – add ‘per course’ after $82.58. Consider rewriting last bullet point – just say how exactly the findings informed the design of DiaDeM and the definitive trial and how that would help going forward.

3. Intro – get an in-depth discussion on the evolution and definitions of multiple conditions in the literature to choose ‘multi-morbidity’ if that’s the case. Please don't use various terms interchangeably. A survey of the global literature on what we know already in terms of modelling cost-effectiveness of multi-morbidity, and what gaps this manuscript would fill in (beyond that it is the first study in Pakistan) would be helpful to make the case for this paper.

4. Methods – Explain how you think Bangladesh would benefit from this work despite it was excluded in this analysis (include this in the discussion section). Mention of “optimised” usual care without explaining what it means. Several typos including error in reference links. Address the first major comment when you present the two-way interaction. Critically justify every assumption and choice that you have made so that it is clear and replicable. When a particular data is used, explain where the robustness of this is coming from (e.g. Lines 40-46, p. 4). The assumption about ratio of costs being constant is a big one (Lines 3-4, p. 5) – justify. Perhaps follow some framework, e.g. NICE/Gates reference case, to construct the methods section so that you include all important aspects of reporting, e.g. starting with decision problem, defining PICO, etc. to discount rate.

5. Results – Table 1: What is BCP? Table 3: Consider alternative ways to present this data (in addition to this) so that this becomes more pragmatic.

6. Discussion – consider rewriting as this is the weakest part of the manuscript. I think that the first paragraph is redundant (novel approach is not convincing yet) and several paragraphs thereafter are not really discussions; they are extended analysis and should have been included under the results section. Consider discussing major limitations of the model – endogenous construct but static/independent model input data; immediate implications of combined costs perspectives beyond the need for future research, e.g. on the intervention design and its evaluation; pragmatic use of EVPPI etc. etc. (there are several key limitations!).

7. Conclusion – Consider rewriting – a couple of stand-alone sentences saying what you found and how exactly it informed the design of a new (or adapted) intervention and the trial to evaluate it.

8. Fig 2- Consider re-drawing to make it look more professional.

9. Appendices – provides additional information and generally ok but lacks specific details on several occasions. Particularly, consider providing critical justification when you use any assumption or data.

**Reviewer**	**3**
**Name**	**Kelson, Zoe**
**Affiliation**	**University of Exeter, Mathematics**
**Date**	**11-Mar-2025**
**COI**	**None**

This paper reports the use of an innovative multimorbidity microsimulation model to aid design of the 'Developing and evaluating an adapted behavioural activation intervention for depression and diabetes in South Asia (DiaDeM)' trial investigating a psychological intervention, behavioural activation (BA), on people with comorbid diabetes and depression in Bangladesh and Pakistan.

Reviewer comments:

"Over their lifetime, individuals receiving BA avoid 3.85 years of depression and experience fewer diabetes-related events. BA resulted in an additional 0.27 life years, 0.98 DALYs averted, and reduced healthcare costs by $97 (excluding BA costs). The maximum cost per BA course at which was cost effective is $82.58. " [Abstract]

Can confidence intervals please be included for each point estimate stated in the Abstract?

"A microsimulation model assessed the cost-effectiveness of BA versus usual care in people with comorbid diabetes and depression in Pakistan...

A published meta-analysis informed the BA treatment effect. The model estimated (i) maximum cost of BA which would be cost-effectiveness (headroom analysis) to inform intervention design, and (ii) value of reducing uncertainty around different measures (value of information analysis) to prioritise data collection in the DiaDeM study" [Abstract]

and

"A multimorbidity diabetes and depression decision analytic model was developed to estimate the lifetime cost and health impacts of a behavioural activation intervention in addition to usual care versus usual care alone in Pakistan for individuals with comorbid diabetes and depression"

A suitable approach has been taken by the authors.

"See Error! Reference source not found."

Can References please be corrected throughout the article.

"Patient profiles were built based on a representative population of individuals with diabetes from Pakistan using the Baqai Institute of Diabetology & Endocrinology (BIDE) patient registry containing 28,942 individuals (31). information on disease history and heart rate not captured in the registry were imputed using external data (20, 25, 32). The BIDE register did

not include information on depression, so PHQ-9 scores were estimated for individuals based on the relationship observed between covariates and PHQ-9 in the INDEPENDENT trial (25). Full details of the development of the patient population are available in the appendix and descriptive statistics of the patient population for the analysis are provided in Table A2"

and

"it is necessary to check the number of patients required to achieve convergence of population level results. The model was found to have reasonable convergence after simulating approximately 8000 individuals"

and

"Table A2: Summary of simulated patient profiles used in the model. SD = standard deviation, PHQ-9 = patient health questionnaire-9; HbA1c = haemoglobin A1c; BMI = body mass index; LDL = how density lipoprotein; HDL = high density lipoprotein, Hg = haemoglobin"

Can the authors please clarify if and how uncertainty in patient profiles has been included in the model?

Can the authors please confirm how missing data was handled in the model?

"Table A3: Inputs used in predicting depression scores over time. SE = standard error, OLS = ordinary least squares, GLM = generalised linear model, PHQ-9 = patient health questionnaire-9, HbA1c = haemoglobin A1c, BA = behavioural activation."

Did the authors consider conducting bootstrapping to sample input parameters and generate confidence intervals?

"A targeted review of Pakistani costing studies was conducted to identify costs for the model."

and

"Table A4: Costs of diabetes and depression care. SE = standard error, BA = behavioural activation, MI = myocardial infarction, IHD = ischaemic heart disease"

Can the authors please assess and report on uncertainty of costs (e.g. showing ranges of potential cost values in the table)?

"Appendix A7 presents results for four further sensitivity analyses exploring the impact of different assumptions about the cost of BA: 1) $15 based on the expected resource use from the trial intervention, 2) $65.65 found in an Indian study with intensive compliance efforts (44), 3) $8.60 and 4) $214.10, the lower and upper credible intervals from the headroom analysis respectively."

Insightful and informative one-way sensitivity analyses have been conducted regarding the cost assumptions.

"Cost-effectiveness was assessed using a cost-effectiveness threshold of $183 per DALY averted (38-40)."

Can the authors please consider conducting a probabilistic sampling sensitivity analysis (PSA)?

"Headroom analysis allows us to estimate the maximum price of a treatment at which it remains cost effective for a given level of effectiveness... i.e. we estimated the maximum cost of BA for which the ICER of BA versus usual practice is equal to the cost-effectiveness threshold of 183US$"

and

"The VOI analysis is carried out assuming the headroom cost of BA to maximise uncertainty, with alternative costs of BA used for a scenario analyses. "

An insightful analytical scenario has been modelled.

Were other what-if scenarios considered for exploration by the authors?

"Table 1: Clinical outcomes simulated in the DiaDeM model, covering depression outcomes, diabetes related outcomes, mortality and cause of death. UC = Usual Care, BA = behavioural activation, PHQ-9 = Patient Health Questionnaire-9, MI = myocardial infarction, IHD = ischaemic heart disease, CHF = congestive heart failure, CrI = credible interval."

and

"Table 2: Summary of cost-effectiveness results comparing BA in additional to usual care versus usual care alone. BA = behavioural activation, ICER = incremental cost effectiveness ratio, CrI = credible interval."

Can the authors please clarify how the credible intervals were computed in the methods section?

"Further research using directed acyclic graphs is necessary to fully formalise this approach and to estimate time dependent interactions under weaker assumptions (45, 46). In contrast to other models, our approach considered depression as recurrent, episodic condition instead of single episode which further adds to the reliability of results. Health system costs and out of pocket costs are combined into health care costs in this analysis. A full multi sector analysis would be required to treat these costs as falling on different budgets, consumption in the case of out of pocket costs and the health budget in the case of health system costs (15, 47). This is potentially an important area of further research. It should be noted that EVPPI represents the value of eliminating all uncertainty in a group of parameters. Therefore, it represents an upper bound for the value of research. For the task of approximating the relative value of collecting information on different parameters, EVPPI may be a reasonable approximation. However, comparing EVPPI does not consider the (potentially differential) rates at which uncertainty in a given group of parameters are resolved and how this interacts with trial design. An expected value of sample information

(EVSI) analysis would be required to fully capture this aspect of research design (14). This was not carried out here due to computational and evidentiary challenges (48, 49). As a pragmatic alternative, parameter groups were split into short, medium and long term to capture the impact of trial follow up length, which is a novel approach to addressing the problem. The analysis in this paper does not consider screening or costs involved in identification, therefore the results are implicitly based on the case in which individuals are perfectly identified (all true positives). This also relates to the choice of comparator in the model: usual care. Those who are not identified as having depression will go without any treatment. This no treatment option was not included in the analysis as it was not considered a relevant policy alternative. However, it would be necessary to include in an analysis which included screening which may be relevant for decision makers"

A discussion on the study limitations is provided by the authors.

Thanks for providing a copy of the CHEERS checklist.

VERSION 1 - AUTHOR RESPONSE

**Referees comment**	**Response**	**Action**
**Reviewer: 1 Dr. Nawaraj Bhattarai, Institute of Health & Society, Newcastle University**		
This is a novel piece of interesting work carried out by the researchers in a setting limited by the data and could pave way for further research in this area. I appreciate the methodological approach undertaken and would be nice to see this published.	We appreciate the positive feedback on our research.	No action taken.
1. The authors report that the findings of this study will be used to inform the design of the DiaDeM intervention and trial being conducted in Pakistan and Bangladesh. However, their analyses is reported to focus only in Pakistan, because of data unavailability in Bangladesh. Though these countries may be geographically and culturally similar, there might be differences in diabetic and depression profiles. Could the authors highlight what data was unavailable in Bangladesh that stopped them considering it in this study?	As set out in the paper, the model requires a high level of information to be able to parameterise it for a country, including patient level covariates to inform the risk model and relevant resource use and cost data. Even for Pakistan we were unable to identify all data required and have had to base certain parameters on assumptions	We have added to the discussion about the issues of focusing solely on Pakistan for the analysis (pg11):<br><br>“The analysis was carried out to inform the DiaDeM trial which will be carried out in both Pakistan and Bangladesh. We focused on Pakistan only because of a lack of patient level data and cost data for routine care for people with diabetes, or costs associated with major cardiovascular events. This is an important limitation of our pre-trial

	from observed cross country relationships. In Bangladesh, we were unable to source patient level covariates to describe the diabetes population in the country and could also not identify appropriate cost data around routine costs for diabetic patients or costs associated with major cardiovascular events. As such, we focused our efforts on Pakistan for the modelling, and hoped that inferences drawn in Pakistan would also be appropriate for Bangladesh. We would note, that following the completion of the full study, the model will be parameterised for Bangladesh based on the trial data and further data we have developed with partners over the course of the DiaDeM programme. We have acknowledged the limitation of focusing on Pakistan in the discussion but not provided further	analysis because of potential differences between these countries in costs, health behaviours, care delivery etc. Following completion of the DiaDeM trial, the model will be parameterised for both Bangladesh and Pakistan based on data collected in the trial.”

	detail on what information was missing from Bangladesh as a result of a lack of word count.	
2. The discussion section is well written. However, considering the assumptions in the data, and some of the computations (for eg. disease history and heart rate data sourced externally and imputed, the ratio of diabetic related costs in the UK and Bangladesh (what was the ratio used?) or the PHQ-9 data from another trial- were these populations representative of the diabetic/depressive population studied here?), it would be great to see the authors discussing any limitations arising.<br><br>Also, considering the UK costs in GBP (Though, I have not checked the study referred here) and the costs reported in here in USD, how was the conversion done here? It is reported that costs were inflated to 2020 USD. Could the authors also provide the reference to the inflation indices used or report them somewhere in the supplementary file? Were there no limitations in the cost of care per depressive episode reported from a published source?	Thank you for highlighting these issues and we agree that they could be more extensively addressed in our discussion section around the approaches taken and any assumptions made and the limitations of these. We have added further details of these approaches and assumptions to the manuscript and addressed them briefly in the discussion section. We have attempted to apply the best data available and made clear any assumptions, but note that these are still subject to significant uncertainty and risk of bias.	We have added further details to the discussion section addressing the potential limitations of the methods used (pg11-12):<br><br>"Though there were more data for Pakistan than Bangladesh, there were still important data limitations for the Pakistani context. Where necessary, data from other countries were used to parameterise the model. For example, the PHQ-9 data were from an Indian study and disease history was imputed based on the relationship between risk factors estimated based on UK data. Data quality was also an issue for sources within Pakistan, for example the cost of care per depressive episode was based on a secondary care facility which may not be representative of practice. For the UKPDS diabetes model the risk of events is based on patient characteristics (e.g. age, smoking status, HbA1c etc). These distribution of characteristics in the population was based on Pakistani data, however, the risk equations linking characteristics to

		outcomes was based on longitudinal UK data. These extrapolations are an important limitation of the analysis and highlight the need for long term primary data collection."<br><br>We have added additional text to the technical appendix on the approaches used for adjusting costs across countries and time (pg6):<br><br>"The table below shows the diabetes and depression costs used in the model. All cost inputs are for the Pakistani health system and are denominated in 2020 $USD.<br>The background diabetes care costs for Pakistan came from Gupta et al 2020 which reported results for 2017, these were converted to Pakistani rupee (PKR), inflated to the year 2020 and then converted to USD using World Bank data (12, 13). Costs of other adverse events related to diabetes were not available in Pakistan. Therefore, for other diabetic adverse events, UK costs based on Alva et al 2015 were converted to costs to Pakistani 2020 $USD based on the relationship between the Gupta et al costs and Alva et al costs for background diabetes costs (a ratio of 0.3018 was used)."

3.Cost section, Page 6 line 9. Could you please add a reference to the pilot protocol.	Thank you for highlighting this we have now added a reference.	Added reference pg5.
4. "see Error! Reference source not found" is found in multiple places in the manuscript, probably to do the hyperlinks used. Could you please correct these? Also, the manuscript is not correctly page numbered.	Thank you for highlighting this and apologies for this error having occurred. We have now corrected these links in the updated manuscript	Fixed references and page numbers.
5. In the discussion section, page 12, line 22, where the text reads " In contrast to other models......" could you please provide a reference to these models?	Thank you for highlighting this we have now added references for example models. We have also changed the location and structure of the sentence.	New sentence to discussion (pg11):<br><br>"This paper employed a novel approach to predicting outcomes in depression by modelling individual PHQ-9 scores over time. This can be compared to models which consider a single episode of depression (47-49)"<br><br>Added references:<br>• Kuyken W, Byford S, Taylor RS, Watkins E, Holden E, White K, et al. Mindfulness-based cognitive therapy to prevent relapse in recurrent depression. Journal of consulting and clinical psychology. 2008;76(6):966.<br>• Simon GE, Von Korff M, Ludman EJ, Katon WJ, Rutter C, Unützer J, et al. Cost-

		effectiveness of a program to prevent depression relapse in primary care. Medical Care. 2002;40(10):941-50.<br>• Rodgers M, Asaria M, Walker S, McMillan D, Lucock M, Harden M, et al. The clinical effectiveness and cost-effectiveness of low-intensity psychological interventions for the secondary prevention of relapse after depression: a systematic review. Health Technol Assess. 2012;16(28):1-130.
6. Patient population section, page 5, line 40....the sentence "information on disease history...." starts with lower case.	Thank you for highlighting this we have now corrected in the manuscript.	Added capitalisation.
**Reviewer: 2**<br>**Dr. Subhash Pokhrel, Brunel University London**		
This manuscript describes an economic model to predict costs and benefits, primarily to inform intervention design and a clinical trial of a behavioural activation intervention to address a multiple long-term condition (diabetes-depression) in Pakistan. Whilst this is an important area of research, more work is needed to improve its quality for it to get a recommendation for publication.	We would like to thank the author for his comprehensive review and the suggestions to improve the communication of the research.	No action taken.
1. Co-morbidity or multi-morbidity or multiple long-term conditions (MLTCs)- more research on the evolution of these terms, how they are	Thank you for highlighting this. We have explored	We have added text to the introduction and discussion reflecting this

defined and in which context, needs to be done to choose the right term for diabetes-depression context in the manuscript. Both co-morbidity and multi-morbidity are used interchangeably whilst no discussion around multiple-long term conditions or MLTCs, to which the most recent academic debate is oriented. If it is a co-morbidity model, it is important to identify which one the primary diagnosis was for modelling purposes. If it was a multi-morbidity or MLTC model, see point 2 below.	the literature and discussed this as a team. We now use multiple long-term conditions (MLTCs) throughout the paper. This is because our model, and approach, can most accurately be described as the modelling of depression and diabetes equally. We consider a detailed discussion of comorbidity, multi morbidity and multiple long term conditions beyond the scope of this manuscript but we have added brief text and references to this issue in the introduction and discussion.	issue and trying to improve the precision of our language around the conditions we are modelling and their appropriate joint description throughout. We removed references to “co-morbidity” and “multiple morbidity” from title and abstract<br><br>Changed wording from “multimorbidity” and “comorbidity” throughout the main text:<br><br><u>Introduction (pg2):</u><br><br>“Individuals can experience multiple long term conditions (MLTCs) simultaneously (1, 2). In South Asia the co-occurrence of diabetes and depression is recognised as an important issue (3-5). These conditions can interact in complex ways which can increase morbidity and mortality. MTLCs are increasingly recognised as an important issue globally which requires research (6).<br><br>The ‘Developing and evaluating an adapted behavioural activation intervention for depression and diabetes in South Asia’ (DiaDeM) trial is designed to address the co-existence of these MLTCs (7).”<br><br>“Further, there is limited evidence on BA for the

		treatment of depression in MLTCs, including diabetes (11).”<br><br><u>Methods (pg3):</u><br><br>“A MLTC diabetes and depression decision analytic model was developed to estimate the lifetime cost and health impacts of a behavioural activation intervention in addition to usual care versus usual care alone in Pakistan for individuals with both diabetes and depression (16).”<br><br><u>Discussion (pg10):</u><br><br>“In this paper we developed a novel MLTC model of diabetes and depression to assess the potential benefits of a BA intervention and to carry out a headroom and value of information analyses with the aim of informing the DiaDeM intervention design and trial.”<br><br><u>Conclusion (pg12):</u><br><br>“This is the first study to jointly model depression and diabetes for South Asia and uses novel methods to reflect the diseases and their interactions. BA had the potential to be a cost-effective intervention for treatment patients with both depression and diabetes in Pakistan”

2. Linked to point 1 is the lack of clarity in modelling strategy. It appears that an endogenous construct is allowed (a state of both diabetes and depression is jointly determined) whilst model operationalisation used empirical values of independent impact of one on the other and vice versa (joint impact not determined within the model). Was this the limitation due to availability of data or a significant miss?<br><br>There is no discussion and empirical tests conducted to establish the robustness/validity of this modelling component (a simple sensitivity analysis might not pick this up; you might need to let the model estimate the joint impact).	We are unclear exactly what critique you are making of the modelling approach here and would appreciate greater clarity. From our understanding, you appear to be arguing that we have proposed an endogenous relationship between diabetes and depression but then have had to implement this on the basis of independent data sources. This is in some way correct, and while a large literature does exist around the implications of depression on other diseases, including diabetes, there is a more limited evidence on the relationship between diabetes and depression. Ideally, we would have a long term longitudinal data set capturing depression and diabetes over time, in the country of interest to estimate the joint endogenous relationships between the diseases but unfortunately such a dataset does not exist. Instead, we	We have updated the discussion section to be more explicit (pg11):<br><br>"This model structure also allowed us to use individual patient data to estimate the impact that changes in depressive symptoms (PHQ-9) have on diabetes (through HbA1c). External data were used to inform the impact of diabetes-related complications on rates of new depressive episodes (29). This approach to modelling two-way interactions uses separate data sources to estimate the independent effect of diabetes on depression and vice versa. The endogeneity between the MLTCs is then imposed by the model structure. This makes strong implicit assumptions about the underlying causal structure of disease interactions. A more sophisticated approach would require long term longitudinal data on diabetes and depression outcomes. Careful application of causal inference methods would be required in this case to estimate the time dependent endogenous relationship between the MLTCs (50-52)."

	have had to estimate individual relationships from two sources, which we accept is a significant, but in our view, unavoidable limitation of the modelling approach. We have now added additional discussion of this issue in the discussion section of the manuscript.<br><br>The model was developed with support of clinical experts in both the research team and on the advisory committee. Our approach was discussed in detail and considered suitable although the above discussed limitations were noted. We are unaware, in the absence of suitable data, how we would further test the robustness/validity of the modelling approach. The full DiaDeM study provides the opportunity for us to validate some of the details, but even that will only provide limited follow up while understanding the full	

	appropriateness of the approach would require many years of follow up.	
3. One of the major issues of this manuscript is that it imagines the readers already know everything about multi-morbidity and economic modelling, as it makes numerous assumptions and choices without critical justifications. The UK diabetes model is assumed to work with some adjustments and additional depression modules – we are not told with critical evaluation why that would be the case. Same with other approaches taken - the fact that one single study in India had similar approach does not sufficiently justify this is ok. A thorough discussion about the choice of UK model and what specific attributes make it transferable to Pakistani context is needed. An all-rounded discussion around relevance, applicability and generalisability to answer two questions really: (a) is the way UK model is structured and populated with available input data equally resonate with Pakistani context (in whose opinion)?; and (b) would the same level of the performance of the economic model be expected if implemented for Pakistani contexts?	We appreciate that this is a technical manuscript providing detail on a complex decision analytic model developed to address the cost-effectiveness of the treatment of individuals with depression and diabetes in Pakistan and that some readers may require some additional background information on multi morbidity and economic modelling. For this we have provided additional references in the document rather than add further detail to an already lengthy manuscript.<br><br>In particular, with regards to the use of the UK diabetes model and the INDEPENDENT study, we accept that their use and the potential weaknesses of their use in this context had not been sufficiently addressed in the	We have added references on multi morbidity and economic modelling to the introduction. We have added additional details to the discussion section addressing the use of the UKPDS model and the INDEPENDENT trial data (pg11-12):<br><br>"Though there were more data for Pakistan than Bangladesh, there were still important data limitations for the Pakistani context. Where necessary, data from other countries was used to parameterise the model. For example, the PHQ-9 data was from an Indian study and disease history was imputed based on the relationship between risk factors estimated based on UK data. Data quality was also an issue for sources within Pakistan, for example the cost of care per depressive episode was based on a secondary care facility which may not be representative of practice. For the UKPDS diabetes model the risk of events is based on patient characteristics (e.g. age, smoking status, HbA_{1c} etc). These distribution of characteristics in the

	original manuscript. While it is not possible to give extensive details here of all the considerations, we have attempted to add extra detail to the discussion. In brief, the use of the UKPDS model and the outcomes was discussed extensively within the research team and with the expert advisory group, which include Pakistani researchers expert in diabetes. The choice of outcomes was considered to be appropriate, and in the absence of appropriate outcomes data to calibrate the models to the Pakistani context, the use of Pakistani patient profiles to inform the UK based risk models was considered the best option available. With regards to the INDEPENDENT study, this was a study of a similar patient population in a neighbouring country and was considered the most appropriate study available to inform the	population was based on Pakistani data, however the risk equations linking characteristics to outcomes was based on longitudinal UK data. These extrapolations are an important limitation of the analysis and highlight the need for long term primary data collection.”

	depression component and link to diabetes in Pakistan.	
4. Now the first of the two questions could have been answered through PPI work. It is important to clarify why PPI were not involved at all in the design, conduct, reporting and dissemination of this work. Why is this work still useful for local stakeholders including researchers and end-users of the research findings?	In the original paper we did not sufficiently emphasise that the model was discussed in detail with researchers in the country as part of the research team and the expert advisory group. This advisory group also included patient representatives. While we appreciate the input that PPI can bring to research, it is unclear if further PPI work would have led to significant changes in the outcomes considered in the model, which to our understanding are the key major adverse clinical events in diabetes globally. We do accept though that more extensive PPI work may allow us to refine the model in the future and have added a brief sentence to the discussion about the potential benefits of this. Our belief is that this model at present represents	We have added additional detail to the overview and discussion on how it was produced and how further discussion with PPI and local stakeholders could improve future iterations of it.<br><br>Overview (pg3):<br>"The model was produced in collaboration with local researchers and the international DiaDeM advisory group, which included academics, policy makers and patient representatives."<br><br>Discussion (pg12):<br>"The model could also be improved in future by further dialogue with local stakeholders and patients."

	the best available model for predicting the long term outcomes and costs for individuals with diabetes and depression in Pakistan and as such will be highly relevant to local stakeholders. The model is publicly available for them to use and adapt for future decision problems.	
5. It is not clear how the EVPPI analysis would add value pragmatically? Either a clear discussion is provided with current EVPPI data or this analysis be presented in a more pragmatic way to make the findings practically useful for Pakistani decisionmakers.	We apologise for a lack of clarity in the value of the expected value of partial perfect information (EVPPI) in the manuscript. EVPPI is an established methodology to quantify the value of research and captures the potential health impacts of gathering additional information on particular parameters on the basis of improving future decisions (by reducing decision uncertainty). This analysis was primarily of interest in terms of designing the DiaDeM trial, but the lessons from it will also be relevant to other	We have added detail to the discussion section on the value of the EVPPI analysis for the research team and more widely (pg10-11):<br><br><u>Discussion</u><br>"The VOI analysis found that there was considerable value in collecting additional information on the different parameters in the model including large value in short term outcomes such as the effect of depression on diabetes ($395.58m) and PHQ-9 time path ($473.3m). These assessments fed into both the intervention and trial design for DiaDeM. This analysis also suggests that there is considerable value in reducing uncertainty about the time path of biomarkers such as HbA_{1c} and BMI. This can provide an estimate of the value of setting up

	research teams and funders considering research in this patient population in Pakistan. We have added details to the discussion in how this information could be used.	longitudinal data collection for each of these measures. This could then be compared against the expected value of other research projects competing for funding (49)”
6. This manuscript is written in a style that is very hard to understand, even for health economists. Consider rewriting several sentences throughout. In addition, only the last paragraph of the discussion section acknowledges some limitations although there are several.	We have attempted to redraft the manuscript to make it easier to understand for health economists and other readers. We accept that we could have had a more extensive discussion of the limitations of the research and have expanded the discussion section of the manuscript to reflect these limitations more thoroughly,	We have altered the manuscript throughout to improve readability and have added a more detailed limitations section to the discussion.<br><br><u>Discussion (pg11-12):</u><br>“The analysis was carried out to inform the DiaDeM trial which will be carried out in both Pakistan and Bangladesh. We focused on Pakistan only because of lack of data on patient level and appropriate cost data for routine costs for diabetic patients or costs associated with major cardiovascular events. This is an important limitation of our pre-trial analysis because of potential differences between these countries in costs, health behaviours, care delivery etc. Following the completion of the DiaDeM, the model will be parameterised for both Bangladesh and Pakistan based on the data collected in the trial.<br><br>Though there was more data for Pakistan than

		Bangladesh, there were still important data limitations for the Pakistani context. Where necessary, data from other countries was used to parameterise the model. For example, the PHQ-9 data was from an Indian study and disease history was imputed based on the relationship between risk factors estimated based on UK data. Data quality was also an issue for sources within Pakistan, for example the cost of care per depressive episode was based on a secondary care facility which may not be representative of practice. For the UKPDS diabetes model the risk of events is based on patient characteristics (e.g. age, smoking status, HbA_{1c} etc). These distribution of characteristics in the population was based on Pakistani data, however the risk equations linking characteristics to outcomes was based on longitudinal UK data. These extrapolations are an important limitation of the analysis and highlight the need for long term primary data collection. The model could also be improved in future by dialogue with local stakeholders and patients. "
1.Abstract –<br>co-morbidity and multi-morbidity are used interchangeably.	Thank you for these detailed comments, we	Updated abstract (pg1):<br><br>**"Objectives**

"BA+usual care" vs usual care, rather than BA versus usual care.<br><br>State key HE parameters: perspectives, time horizon, discount rate, etc.<br><br>Expand abbreviations while used for the first time.<br><br>Correct typo.<br><br>Consider rewriting conclusion: focus on how exactly the work informed the design of DiaDeM intervention and the definitive trial (which aspect of the findings directly led to that?).	have updated the abstract as suggested.	The 'Developing and evaluating an adapted behavioural activation intervention for depression and diabetes in South Asia (DiaDeM)' trial investigates a psychological intervention, behavioural activation (BA), on people with both diabetes and depression in Bangladesh and Pakistan. This study aimed to aid the intervention and trial design.<br>**Design**<br>This was a modelling study using microsimulation to assess the intervention's cost-effectiveness. Diabetes was modelled using the UK Prospective Diabetes Study (UKPDS) model based on Pakistani patients and depression was modelled using Patient Health Questionnaire-9 trajectories allowing for multiple depressive episodes. It was assumed that diabetes-related adverse events increased depression recurrence, while depression impacted HbA1c, increasing diabetes-related events.<br>The model estimated (i) maximum cost of BA which would be cost-effectiveness (headroom analysis) to inform intervention design, and (ii) value of reducing uncertainty around different measures

		(value of information analysis) to prioritise data collection in the DiaDeM study<br>**Setting**<br>Analysis was conducted from a Pakistani healthcare perspective over a lifetime with costs and outcomes discounted at 3%.<br>**Interventions**<br>BA plus usual care was compared against usual care. BA involved six sessions by a trained (non-mental health) facilitator. The usual care comparator was the prevailing mix of pharmacological and non-pharmacological treatments used in Pakistan.<br>**Primary and Secondary Outcome Measures**<br>The primary outcome was disability-adjusted life-years (DALYs). Secondary outcomes included life years, healthcare costs and the rate of depression and diabetes-related events.<br>**Results**<br>Over their lifetime, individuals receiving BA plus usual care avoid 3.85 years of depression and experience fewer diabetes-related events. BA plus usual care resulted in an additional 0.27 life years, 0.98 DALYs averted, and reduced healthcare costs by $97 (excluding BA costs). The maximum cost per BA course at which was cost effective is $82.58. Value of

		information analysis found the most important measures to include in the trial are impact of depression on diabetes and PHQ-9 over time.<br>**Conclusions**<br>This is the first model to jointly model depression and diabetes for South Asia and uses novel methods to reflect the diseases and inform intervention and trial design. This evidence has helped to inform the design of the DiaDeM intervention and the trial to evaluate it.”
2. Key messages – add ‘per course’ after $82.58. Consider rewriting last bullet point – just say how exactly the findings informed the design of DiaDeM and the definitive trial and how that would help going forward.	Thank you for your suggestion. The “key messages” section has been removed on the suggestion of the editor.	No action taken
3. Intro – get an in-depth discussion on the evolution and definitions of multiple conditions in the literature to choose ‘multi-morbidity’ if that’s the case. Please don't use various terms interchangeably.<br><br>A survey of the global literature on what we know already in terms of modelling cost-effectiveness of multi-morbidity, and what gaps this manuscript would fill in (beyond that it is the first study in Pakistan) would be helpful to make the case for this paper.	We have addressed this comment above, the evolution of the terminology is beyond this paper but we have cited the literature on this as a signpost for interested readers.<br><br>A review of the literature around cost-effectiveness of multi-morbidity is beyond the scope of this study where the focus is on only two conditions. We have noted previous work	We have added references to the introduction on terminology and added details on previous cost-effectiveness studies of depression and diabetes (pg2):<br><br>“Further, there is limited evidence on BA for the treatment of depression in MLTCs, including diabetes (12). This study will add to the growing literature on cost effectiveness in the context of MLTCs (7, 13, 14).”<br><br>References added:<br>Banstola A, Anokye N, Pokhrel S. The economic

	considering diabetes and depression jointly, which is limited. With regards to wider literature on cost-effectiveness of multimorbidity we have added a reference to the protocol for the reviewer's own planned systematic review around multimorbidity and economic impacts.	burden of multimorbidity: Protocol for a systematic review. Plos one. 2024;19(5):e0301485.<br><br>Emmert-Fees KMF, Laxy M, Patel SA, Singh K, Poongothai S, Mohan V, et al. Cost-Effectiveness of a Collaborative Care Model Among Patients With Type 2 Diabetes and Depression in India. Diabetes Care. 2022;46(1):11-9.<br><br>Kearns B, Rafia R, Leaviss J, Preston L, Brazier JE, Palmer S, et al. The cost-effectiveness of changes to the care pathway used to identify depression and provide treatment amongst people with diabetes in England: a model-based economic evaluation. BMC Health Serv Res. 2017;17(1):78.
4. Methods – Explain how you think Bangladesh would benefit from this work despite it was excluded in this analysis (include this in the discussion section).<br><br>Mention of "optimised" usual care without explaining what it means.<br><br>Several typos including error in reference links.<br><br>Address the first major comment when you present the two-way interaction.<br><br>Critically justify every assumption and choice that you have made so that it is clear and replicable.<br><br>When a particular data is used, explain where the robustness of this is coming from (e.g. Lines 40-46, p. 4).	We agree that more detail is needed on the potential value of this work for Bangladesh, although we feel this is better placed in the discussion rather than methods section.<br><br>We have added further details of what optimised care represented in the planned study.	Added text:<br><br><u>Methods (pg3):</u><br>"The usual care comparator is the prevailing mix of pharmacological and non-pharmacological treatments used in Pakistan, "optimisation" in this case is the provision of an information leaflet with details for accessing care locally (25)."<br><br><u>Discussion (pg11-12):</u><br>"The analysis was carried out to inform the DiaDeM trial which will

The assumption about ratio of costs being constant is a big one (Lines 3-4, p. 5) – justify.<br><br>Perhaps follow some framework, e.g. NICE/Gates reference case, to construct the methods section so that you include all important aspects of reporting, e.g. starting with decision problem, defining PICO, etc. to discount rate.	We have added details on assumptions and choices made but we do not feel it is possible to critically justify every decision made in a complex economic model in the limited word count available.<br><br>Similarly we have added additional details on data, but we do not feel it is possible or appropriate to justify the robustness at each point.<br><br>We agree that the approach to costing and assumptions have not been sufficiently described and critiqued and this has been raised by other reviewers. We have now provided further detail and discussion around this.<br><br>The work has been conducted in line with the Gates reference case for economic evaluation and we have completed a CHEERS checklist to demonstrate that we have reported the results in line with	be carried out in both Pakistan and Bangladesh. We focused on Pakistan only because of lack of data on patient level and appropriate cost data for routine costs for diabetic patients or costs associated with major cardiovascular events. This is an important limitation of our pre-trial analysis because of potential differences between these countries in costs, health behaviours, care delivery etc. Following the completion of the DiaDeM, the model will be parameterised for both Bangladesh and Pakistan based on the data collected in the trial.<br><br>Though there was more data for Pakistan than Bangladesh, there were still important data limitations for the Pakistani context. Where necessary, data from other countries was used to parameterise the model. For example, the PHQ-9 data was from an Indian study and disease history was imputed based on the relationship between risk factors estimated based on UK data. Data quality was also an issue for sources within Pakistan, for example the cost of care per depressive episode was based on a secondary care facility which may

	the most widely used guidelines for cost-effectiveness.	not be representative of practice. For the UKPDS diabetes model the risk of events is based on patient characteristics (e.g. age, smoking status, HbA1c etc). These distribution of characteristics in the population was based on Pakistani data, however the risk equations linking characteristics to outcomes was based on longitudinal UK data. These extrapolations are an important limitation of the analysis and highlight the need for long term primary data collection. The model could also be improved in future by dialogue with local stakeholders and patients.”
5. Results – Table 1: What is BCP?<br><br>Table 3: Consider alternative ways to present this data (in addition to this) so that this becomes more pragmatic.	Thank you for highlighting “BCP” this was old terminology which we have now removed.<br><br>We are unclear about what the reviewer means by “more pragmatic”.	Table 1: removed “BCP” and replaced by “UC” i.e. usual care.
6. Discussion – consider rewriting as this is the weakest part of the manuscript.<br><br>I think that the first paragraph is redundant (novel approach is not convincing yet) and<br><br>several paragraphs thereafter are not really discussions; they are extended analysis and should have been included under the results section.<br><br>Consider discussing major limitations of the model – endogenous construct but static/independent model input data;	We have added to the discussion and provided further details.<br><br>We would argue that our approach is novel, and this has been supported by the other reviewers.	Added text/rewritten discussion (pg11-12):<br><br>“The analysis was carried out to inform the DiaDeM trial which will be carried out in both Pakistan and Bangladesh. We focused on Pakistan only because of lack of data on patient level and appropriate cost data for routine

immediate implications of combined costs perspectives beyond the need for future research, e.g. on the intervention design and its evaluation;<br><br>pragmatic use of EVPPI etc. etc. (there are several key limitations!).	We agree that the limitations section was too brief and have substantially extended this to reflect the comments of this and other reviewers.<br><br>We are unclear what the reviewer means by implications of combined cost perspectives.<br><br>We have address the issue of EVPPI above.	costs for diabetic patients or costs associated with major cardiovascular events. This is an important limitation of our pre-trial analysis because of potential differences between these countries in costs, health behaviours, care delivery etc. Following the completion of the DiaDeM, the model will be parameterised for both Bangladesh and Pakistan based on the data collected in the trial.<br><br>Though there was more data for Pakistan than Bangladesh, there were still important data limitations for the Pakistani context. Where necessary, data from other countries was used to parameterise the model. For example, the PHQ-9 data was from an Indian study and disease history was imputed based on the relationship between risk factors estimated based on UK data. Data quality was also an issue for sources within Pakistan, for example the cost of care per depressive episode was based on a secondary care facility which may not be representative of practice. For the UKPDS diabetes model the risk of events is based on patient characteristics (e.g. age, smoking status, HbA_{1c} etc). These

		distribution of characteristics in the population was based on Pakistani data, however the risk equations linking characteristics to outcomes was based on longitudinal UK data. These extrapolations are an important limitation of the analysis and highlight the need for long term primary data collection. The model could also be improved in future by dialogue with local stakeholders and patients. "<br><br>"The VOI analysis found that there was considerable value in collecting additional information on the different parameters in the model including large value in short term outcomes such as the effect of depression on diabetes (\$395.58m) and PHQ-9 time path (\$473.3m). These assessments fed into both the intervention and trial design for DiaDeM. This analysis also suggests that there is considerable value in reducing uncertainty about the time path of biomarkers such as. HbA_{1c}, BMI etc. This can provide an estimate of the value of setting up longitudinal data collection for each of these measures. This could then be compared against the value of other research projects

		competing for funding (48).”
7. Conclusion – Consider rewriting – a couple of stand-alone sentences saying what you found and how exactly it informed the design of a new (or adapted) intervention and the trial to evaluate it.	We have updated the conclusions to make it clearer about the key findings of the research.	Rewritten conclusion (pg12):<br><br>“We found that BA had the potential to be a cost-effective intervention for the treatment of patients with both depression and diabetes in Pakistan, improving morbidity and mortality and reducing costs, although efforts must be made to keep the cost low and uncertainty remains over the impact of depression on diabetes and trajectory of depression. This evidence has helped to inform the design of the DiaDeM intervention by highlighting the value of collecting evidence on the impact of depression on diabetes and the time path of PHQ-9 scores. The model developed will be used to estimate the cost-effectiveness of the intervention following completion of the definitive trial.”
8. Fig 2- Consider re-drawing to make it look more professional.	Thank you for the suggestion. We agree and have redrawn the figure.	Redrawn figure 2.
9. Appendices – provides additional information and generally ok but lacks specific details on several occasions. Particularly, consider providing critical justification when you use any assumption or data.	We have added additional detail to the appendices. We do not however believe it is possible to provide critical justification of	We have added additional text to the technical appendix on the approaches used for adjusting costs across countries and time (pg6):

	every decision made throughout the study with regard to the data and assumptions used.	“The table below shows the diabetes and depression costs used in the model. All cost inputs are for the Pakistani health system and are denominated in 2020 $USD.<br>The background diabetes care costs for Pakistan came from Gupta et al 2020 which reported results for 2017, these were converted to Pakistani rupee (PKR), inflated to the year 2020 and then converted to USD using World Bank data (12, 13). Costs of other adverse events related to diabetes were not available in Pakistan. Therefore, for other diabetes these costs, UK costs based on Alva et al 2015 were converted to costs to Pakistani 2020 $USD based on the relationship between the Gupta et al costs and Alva et al costs for background diabetes costs (a ratio of 0.3018 was used).”
**Reviewer: 3**<br>**Prof. Zoe Kelson, University of Exeter**		
"Over their lifetime, individuals receiving BA avoid 3.85 years of depression and experience fewer diabetes-related events. BA resulted in an additional 0.27 life years, 0.98 DALYs averted, and reduced healthcare costs by $97 (excluding BA costs). The maximum cost per BA course at which was cost effective is $82.58. " [Abstract]<br><br>Can confidence intervals please be included for each point estimate stated in the Abstract?	We have now added credible intervals to the outcomes in the abstract. These are considered “credible” rather than “confidence” intervals because of the implicitly Bayesian interpretation of uncertainty required for	Abstract (pg1):<br><br>“Over their lifetime, individuals receiving BA plus usual care avoid 3.2 (3.8, 2.7) years of mild depression and experience fewer diabetes-related events. BA plus usual care resulted in an additional 0.27 (0.03, 0.52) life years, 0.98 (1.86, 0.45) DALYs averted, and had

	probabilistic sensitivity analysis.	incremental healthcare costs of -$97 (-$517, $142), excluding BA costs. The maximum cost per BA course at which the intervention was cost effective compared to enhanced usual care was $83 ($9, $214). Value of information analysis found the most important measures to include in the trial are impact of depression on diabetes and PHQ-9 over time.”
"See Error! Reference source not found."<br><br>Can References please be corrected throughout the article.	Apologies for these errors in the original uploaded manuscript. These have now been corrected in the updated manuscript.	Fixed references
"Patient profiles were built based on a representative population of individuals with diabetes from Pakistan using the Baqai Institute of Diabetology & Endocrinology (BIDE) patient registry containing 28,942 individuals (31). information on disease history and heart rate not captured in the registry were imputed using external data (20, 25, 32). The BIDE register did not include information on depression, so PHQ-9 scores were estimated for individuals based on the relationship observed between covariates and PHQ-9 in the INDEPENDENT trial (25). Full details of the development of the patient population are available in the appendix and descriptive statistics of the patient population for the analysis are provided in Table A2"<br>and<br>"it is necessary to check the number of patients required to achieve convergence of population level results. The model was found to have reasonable convergence after simulating approximately 8000 individuals"<br>and<br>"Table A2: Summary of simulated patient profiles used in the model. SD = standard deviation, PHQ-9 = patient health questionnaire-9; HbA1c =	Thank you for this comment and we are happy to provide further information about the approach to uncertainty in the patient profiles, but also, more importantly variability.<br><br>As described patient profiles for the use in the individual patient simulation model were based on individual patients from the BIDE registry supplemented with evidence from the INDEPENDENT trial and other studies	We have added some text around the imputation and the approach to reflecting uncertainty and variability in the analysis to the manuscript.<br><br><u>Patient population (pg4-5):</u><br>“Patient profiles were built based on a representative population of individuals with diabetes from Pakistan using the Baqai Institute of Diabetology & Endocrinology (BIDE) patient registry containing 28,942 individuals (35). Information on disease history and heart rate not captured in the registry were imputed using external data (14,

haemoglobin A1c; BMI = body mass index; LDL = how density lipoprotein; HDL = high density lipoprotein, Hg = haemoglobin"<br><br>Can the authors please clarify if and how uncertainty in patient profiles has been included in the model?	for covariates not included in the registry (depression, measured by PHQ-9, disease history and health rate). For non-included covariates, predictions were based on observed relationships between known covariates and the covariates of interest from the other data sources. All these predictions allowed for uncertainty in the prediction model. Further, missing covariates were imputed using multiple imputation by chain equations, which produces imputations accounting for the underlying uncertainty. This allowed for us to account for uncertainty in the unobserved covariates in the patient population. To account for variability in patients, we randomly sampled patient profiles from the created data set. It was assumed that the joint distribution of patient characteristics in	29, 36). The BIDE register did not include information on depression, so PHQ-9 scores were estimated for individuals based on the relationship observed between covariates and PHQ-9 in the INDEPENDENT trial (29), with uncertainty in predictions captured. Further, missing covariates were imputed using multiple imputation by chain equations (MICE), which produces imputations accounting for the underlying uncertainty. To account for variability in patients, we randomly sampled patient profiles from the created data set. It was assumed that the joint distribution of patient characteristics in our patient profile data set (based on BIDE and other sources) represented the joint distribution in the population of interest. Full details of the development of the patient population are available in the appendix and descriptive statistics of the patient population for the analysis are provided in Table A2.“

	BIDE represented the joint distribution in the population of interest.	
Can the authors please confirm how missing data was handled in the model?	Above we have briefly described how missing data in the patient profiles was handled. Data for the estimation of other parameters in the model were sourced from different studies with assumptions required to facilitate exchangeability described.	Patient population (pg4-5):<br><br>"The BIDE register did not include information on depression, so PHQ-9 scores were estimated for individuals based on the relationship observed between covariates and PHQ-9 in the INDEPENDENT trial (29). The uncertainty in the estimated prediction models was reflected in the results (see section "Generating model predictions"). Further, missing covariates were imputed using multiple imputation by chain equations (MICE), which produces imputations accounting for the underlying uncertainty. This allowed for us to account for uncertainty in the unobserved covariates in the patient population. To account for variability in patients, we randomly sampled patient profiles from the created data set. It was assumed that the joint distribution of patient characteristics in BIDE represented the joint distribution in the population of interest. Full details of the development of the patient population are available in the appendix and descriptive statistics of the patient population

		for the analysis are provided in Table A2."
"Table A3: Inputs used in predicting depression scores over time. SE = standard error, OLS = ordinary least squares, GLM = generalised linear model, PHQ-9 = patient health questionnaire-9, HbA1c = haemoglobin A1c, BA = behavioural activation."<br><br>Did the authors consider conducting bootstrapping to sample input parameters and generate confidence intervals?	For this analysis the methods used (OLS and GLM) allow us to reflect the uncertainty parametrically and provide confidence intervals without the need for bootstrapping (as is standard in cost effectiveness analysis these confidence intervals are interpreted as Bayesian credible intervals). We could potentially have additionally bootstrapped to reflect variability in the data set in the uncertainty estimates but we did not consider it necessary in this case and believe that the parametric approach will appropriately reflect the uncertainty in our estimates.	No change to manuscript.
"A targeted review of Pakistani costing studies was conducted to identify costs for the model." and<br>"Table A4: Costs of diabetes and depression care. SE = standard error, BA = behavioural activation, MI = myocardial infarction, IHD = ischaemic heart disease"<br><br>Can the authors please assess and report on uncertainty of costs (e.g. showing ranges of potential cost values in the table)?	Thank you for highlighting this. The models used for most of the cost data come from Alva et al 2015. This model computed total costs for each diabetes event using an inpatient	Updated supplementary material (pg6):<br><br><u>A4 Costs of diabetes and depression care</u><br>"Note that costs in Alva et al 2015 are composed of inpatient and outpatient costs, with the probability of outpatient costs

	and outpatient cost regression. There was also an additional logistic regression which estimated the probability of requiring inpatient care. Because the costs presented in Table A4 are an aggregate of each of these three models, standard errors cannot be reported. However, in the probabilistic sensitivity analysis we did reflect this uncertainty. We have added text to explain this.	determined by a logistic regression. Therefore, standard errors could not be quoted for these aggregated inputs. However, the component inpatient, outpatient and logistic regression models were all estimated using generalised linear models and so standard errors were computed for each parameter. The uncertainty in these models was reflected in the probabilistic sensitivity analysis (PSA), see section "Generating model predictions" in the main text."
"Cost-effectiveness was assessed using a cost-effectiveness threshold of $183 per DALY averted (38-40)."<br>Can the authors please consider conducting a probabilistic sampling sensitivity analysis (PSA)?	Thank you for pointing this out. We did conduct a probabilistic sensitivity analysis, which reflected parametric uncertainty in all model parameters. But we mistakenly omitted this from the paper. This has now been added.<br><br>We have not considered the uncertainty in the cost-effectiveness threshold as this is a decision variable separate to the model. It would not be standard practice to consider uncertainty in the cost-effectiveness threshold	We have added the following text to the main paper:<br><br>Generating model predictions (pg6):<br>"To reflect uncertainty in model inputs a probabilistic sensitivity analysis (PSA) was carried out by drawing 1000 times from the sampling distribution of each input parameter (15). Outcomes (e.g. life years, DALYs etc) were computed for each combination of input values resulting in a posterior distribution for each outcome. Point estimates were computed from the mean of these distributions and credible intervals by computing the relevant quantiles."

	probabilistically although we do accept that there is uncertainty around the appropriate choice of cost-effectiveness threshold in this setting. However, there is no easy way to reflect the uncertainty around the estimate used (we have briefly discussed this with the authors of the paper on which this was based who are colleagues). Further the key uncertainties around threshold choice are likely to be structural rather than parametric	
"Headroom analysis allows us to estimate the maximum price of a treatment at which it remains cost effective for a given level of effectiveness... i.e. we estimated the maximum cost of BA for which the ICER of BA versus usual practice is equal to the cost-effectiveness threshold of 183US$" and<br>"The VOI analysis is carried out assuming the headroom cost of BA to maximise uncertainty, with alternative costs of BA used for a scenario analyses. "<br>An insightful analytical scenario has been modelled.<br><br>Were other what-if scenarios considered for exploration by the authors?	We have reported all of the scenarios which were conducted within the manuscript and appendix. Scenarios were chosen based on their significance for informing the decision and the ability to accurately reflect the underlying uncertainty in the model. Unfortunately, as a result of the computational requirements, it was not possible	No change to manuscript.

	to run fully comprehensive sensitivity and scenario analyses of all potential issues and uncertainties.	
"Table 1: Clinical outcomes simulated in the DiaDeM model, covering depression outcomes, diabetes related outcomes, mortality and cause of death. UC = Usual Care, BA = behavioural activation, PHQ-9 = Patient Health Questionnaire-9, MI = myocardial infarction, IHD = ischaemic heart disease, CHF = congestive heart failure, CrI = credible interval."<br>and<br>"Table 2: Summary of cost-effectiveness results comparing BA in additional to usual care versus usual care alone. BA = behavioural activation, ICER = incremental cost effectiveness ratio, CrI = credible interval."<br><br>Can the authors please clarify how the credible intervals were computed in the methods section?	These credible intervals were computed based on quantiles of the output from the probabilistic sensitivity analysis calculated as part of the study- that is they represent the 2.5% and 97.5% percentiles of the outputs of the PSA. We have added text to the manuscript to clarify this.	Text added to manuscript:<br><br>Generating model predictions (pg6):<br><br>"To reflect uncertainty in model inputs a probabilistic sensitivity analysis (PSA) was carried out by drawing 1000 times from the sampling distribution of each input parameter (15). Outcomes (e.g. life years, DALYs etc) were computed for each combination of input values resulting in a posterior distribution for each outcome. Point estimates were computed from the mean of these distributions and credible intervals by computing the relevant quantiles."
